# Kinesiophobia and associated factors among patients after cardiac surgery under cardiopulmonary bypass: a cross-sectional study

**DOI:** 10.3389/fpsyt.2025.1584789

**Published:** 2025-07-14

**Authors:** Min You, Qianqian Mou, Xiaotao Cao, Meng Luo, Yi Song, Qingqing Li, Huiyun Hu, Jianping Song

**Affiliations:** ^1^ Clinical Trial Center, West China Hospital, Sichuan University, Chengdu, Sichuan, China; ^2^ West China School of Nursing, Sichuan University, Chengdu, Sichuan, China; ^3^ Nursing Department, The Second Affiliated Hospital of Zhejiang University School of Medicine, Hangzhou, Zhejiang, China

**Keywords:** cardiopulmonary bypass, cardiac surgery, kinesiophobia, associated factors, postoperative rehabilitation

## Abstract

**Background:**

Kinesiophobia is a significant psychological factor that affects the early ambulation of postoperative patients, and is especially prevalent among postoperative cardiac patients. Nonetheless, few studies have explored this phenomenon in patients following cardiac surgery under cardiopulmonary bypass.

**Objectives:**

The primary objective of this study was to investigate the prevalence of kinesiophobia and its contributing factors in patients after cardiac surgery under cardiopulmonary bypass, to inform the development of targeted intervention strategies to address kinesiophobia, enhance patient motivation and adherence to early activities, and optimize postoperative rehabilitation outcomes.

**Methods:**

A cross-sectional survey was conducted, assessing patients via questionnaires, including the Tampa Scale for Kinesiophobia Heart, Exercise Self-Efficacy Scale, D Personality Scale-14, Pain Catastrophizing Scale, and Family Care Index Questionnaire. The *χ2 test*, *Fisher’s exact tes*t, and *Mann-Whitney U test* were used for univariate analysis, and binary Logistic stepwise regression analysis was adopted for multivariate analysis. The difference was considered statistically significant at *P*<0.05.

**Results:**

The study encompassed a total of 293 patients, of whom 45.73% (134 patients) exhibited kinesiophobia. Binary logistic regression identified the following risk factors: being accompanied by a nursing assistant (*OR* = 9.009, *P* < 0.001), longer cardiopulmonary bypass duration (*OR* = 1.005, *P* < 0.05), delayed first postoperative ambulation (*OR* = 4.031, *P* < 0.05), Type D personality (*OR* = 2.582, *P* < 0.01), heightened safety concerns (*OR* = 8.270, *P* < 0.05), and pain catastrophizing (*OR* = 4.253, *P* < 0.001). Conversely, family function and exercise self-efficacy mitigated kinesiophobia (*P* < 0.05).

**Conclusions:**

Kinesiophobia is highly prevalent and multifactorial in post-cardiopulmonary bypass patients. Healthcare providers should prioritize patients with the above risk profiles, while also establishing financial/emotional support systems, improving patient-family communication, optimizing acute pain management, implementing Enhanced Recovery After Cardiac Surgery protocols, and leveraging technology to design rehabilitation programs. These measures may reduce kinesiophobia and maximize the benefits of early ambulation.

## 1 Introduction

Cardiovascular disease (CVD) has risen significantly due to population aging and lifestyle changes, driving increased global morbidity and mortality. Over the past three decades, the global prevalence of CVD has increased by 93% (1), affecting hundreds of millions patients, while mortality rates have climbed by approximately 54%, resulting in 17.5 million annual deaths and imposing a substantial burden on global health and economies (1, 2). Cardiac surgery with cardiopulmonary bypass (CPB) has become a cornerstone of CVD treatment, effectively alleviating symptoms, slowing disease progression, and improving survival rates. CPB involves using specialized equipment to temporarily replace heart and lung functions, enabling surgeons to repair or replace valves and vessels during cardiac arrest (3). As techniques advance, surgical success rates have risen, complications have declined, and procedural volumes have increased. Globally, over 2 million cardiac surgeries are performed annually, most requiring CPB support (4).

Despite its benefits, CPB involves complex non-physiological processes, and patients face elevated risks of complications such as atelectasis, delirium, and low cardiac output syndrome (5). Respiratory dysfunction is particularly consequential, driven by CPB-induced systemic inflammation, ischemia-reperfusion injury, and alveolar-capillary damage from non-pulsatile flow and hemodilution. These mechanisms impair pulmonary perfusion, causing ventilation-perfusion mismatch and reduced lung compliance, often manifesting as ARDS, atelectasis, or prolonged mechanical ventilation—the leading morbidity in on-pump surgery (6, 7). Critically, respiratory complications independently predict prolonged ICU stays, higher mortality, and delayed recovery (8, 9), exacerbating perioperative risks and extending hospitalization. Which not only increases the pain of patients, delays their functional recovery but also prolongs their hospital stay. Therefore, promoting the physical rehabilitation of postoperative patients and improving their quality of life have become important goals in treating CVD.

A large amount of clinical data shows that early postoperative ambulation is vital for recovery, enhancing cardiopulmonary endurance, reducing stress responses, shortening hospital stays, and lowering costs (10, 11). However, although early ambulation has numerous benefits, in clinical practice, it has been found that many patients experience kinesiophobia after surgery, resulting in a low participation rate and poor compliance in their early ambulation (12, 13). Kinesiophobia is an exaggerated and irrational fear of physical activity due to concerns about pain or injury, which can lead to avoidance behavior and hinder rehabilitation (14, 15). Kinesiophobia is a psychological stress response that occurs during the disease treatment process. It has been reported that the incidence rate of kinesiophobia in patients after cardiac surgery ranges from 20% to 83%, and largely reduces the enthusiasm and compliance of patients in early rehabilitation (14, 16). Kinesiophobia diminishes participation in early mobilization (17), reduces 6-minute walking distance (18), and compromises rehabilitation outcomes and quality of life (19). For CPB patients, postoperative pain, fatigue, and weakness may exacerbate cognitive biases and psychological insecurity, further hindering ambulation.

Examining kinesiophobia through stress system process theory (15) clarifies how psychological stressors and imbalanced factors disrupt rehabilitation. This study investigates kinesiophobia’ s determinants in post-CPB patients, aiming to inform targeted interventions that address stressors and improve recovery trajectories.

## 2 Methods

### 2.1 Design and study population

This is a cross-sectional study that utilized a convenience sampling method to select subjects. Patients with cardiac disease who were admitted to the Cardiovascular Surgery Intensive Care Unit of a hospital between February 2023 and December 2023 were sequentially included as study subjects. The individuals in this study satisfied specific criteria (1) Aged 18–70 years old; (2) Underwent heart or large vessel surgery under CPB for the first time and met postoperative ambulation criteria including consciousness, normal vital signs, blood oxygen saturation ≥ 95%, absence of vasoactive drugs or recent dose increase, pain numerical score(NRS)≤ 4 points, and muscle strength ≥ 4; (3) Possessed normal communication and literacy abilities, could fill in questionnaires, and gave informed consent for voluntary participation in the study. Exclusion criteria included a history of cognitive impairment or mental illness, severe physical activity disorder, or withdrawal from the study.

### 2.2 Sample

In quantitative nursing science studies that involve multiple variables, it is a standard practice to ensure the sample size is at least 10 times the number of variables (20). Given that this study anticipated 18 variables, considering a 20% attrition rate, we ultimately determined that a minimum sample size of 225 cases would be necessary for robust analysis [18 * 10 = 180, 180/(1 - 0.20) = 225].

### 2.3 Data collection

Given the heterogeneity in participants’ primary diagnoses, disease severity, and surgical procedures, no consensus exists regarding the optimal timing for early postoperative ambulation following cardiac surgery. Consequently, this study determined the timing of questionnaire administration based on functional readiness for ambulation (achievement of mobilization criteria). Informed by rehabilitation literature and clinical consensus, participants were surveyed before their first postoperative ambulation within postoperative days 2-7. Before the survey, researchers explained the survey’s purpose, obtained informed consent, and provided assistance as needed. During the survey, patients filled out the questionnaire independently, and those who were unable to do so were assisted by the researcher according to the patient’s intention. Any doubts about the questionnaire were clarified using standardized language. Each questionnaire took 15–20 minutes to complete and was collected immediately to ensure accuracy.

### 2.4 Measurements

General information questionnaire included three parts: ① Socio-demographic factors: gender, age, educational level, marital status, family per capita monthly income, medical expense payment method, and companion; ② Clinical factors: previous history of cardiovascular diseases, type of surgery, operative time, CPB duration, pain level, time for the first postoperative ambulation; ③ Psychological factors: degree of safety concern, exercise self-efficacy, type D personality, family function, pain catastrophizing.

The Tampa Scale for Kinesiophobia Heart (TSK-SV Heart) was adapted by Dr. Bäck et al. (21) in 2012. The scale includes 4 dimensions and 17 entries, including fear of injury, fear of activity, avoidance of exercise, and dysfunction. The entries were rated on a 4-point Likert scale, ranging from 1 for “completely disagree” to 4 for “completely agree”, with entries 4, 8, 12, and 16 being reversed scores. The overall score ranged from 17 to 68 points, with a score above 37 indicating kinesiophobia. Higher scores reflect a greater degree of kinesiophobia. The *Cronbach’s alpha* coefficient was 0.755 in this study.

Exercise Self-Efficacy Scale (ESES) was developed by Kroll et al. (22) to measure individuals’ confidence in adhering to exercise. The scale is a unidimensional scale consisting of 10 items, each of which is rated on a 4-point Likert scale, ranging from 1 for “completely inconsistent” to 4 for “completely consistent.” The total score on the scale was 10 to 40 points, with higher scores indicating greater confidence in exercise adherence. The *Cronbach’s alpha* coefficient of this scale in this study was 0.884.

Type Personality Scale-14 (DS-14) was compiled by Dutch psychologists Denollet et al. (23) to measure patients’ personality types, consisting of 2 latitudes, negative affect, and social inhibition, with a total of 14 items, which were scored on a Likert 5-point scale, ranging from 0 for “completely inconsistent” to 4 for “completely consistent,” with items 1 and 3 being reverse-scored. The total score for each subscale ranges from 0 to 28 points. A score of “negative affectivity ≥ 10 points and social inhibition ≥ 10 points” indicated a Type D personality. The *Cronbach’s alpha* coefficient for this scale was 0.889.

The Chinese Version of the Pain Catastrophizing Scale (PCS), was originally compiled by Sullivan et al. (24) in 1995 and translated into Chinese by Yap. Which consists of 13 items across three dimensions: rumination, exaggeration, and helplessness. Each item is scored on a 5-point Likert scale, ranging from 0 for “never” to 4 for “always”. The total score of the scale ranged from 0 to 52, and a total score on ≥38 indicated the presence of pain catastrophizing. In this study, the *Cronbach’s α* for this scale was 0.975.

The Family APGAR Index Questionnaire, designed by Smilkstein et al. (25) in 1978, evaluates an individual’s satisfaction with family function. The questionnaire measures five dimensions: Adaptation, Partnership, Growth, Affection, and Resolve. Each item is scored on a 3-point Likert scale, with “always” scored as 2, “sometimes” as 1, and “rarely” as 0. The total score ranges from 0 to 10 points, with higher scores indicating better family function. 0 to 3 indicate severe family dysfunction, 4 to 6 indicates moderate dysfunction and 7 to 10 indicates good family function. The *Cronbach’s alpha* coefficient for this scale was 0.716 in this study.

### 2.5 Ethical and research approvals

This study has been approved by the Ethics Committee of the hospital (IR20220227). All participants consented to participate in this study and provided informed consent in writing. All methods were carried out according to relevant guidelines and regulations.

### 2.6 Statistical analysis

Data were meticulously entered into Excel by two specialists from the research team, and statistical analyses were conducted using SPSS 26.0 software. The analysis included a statistical description, where measurement data were expressed as M ± SD and count data as frequency and composition ratio (%). Non-normally distributed measurement data were expressed by median and quartile [P50 (P25, P75)]. For univariate analysis, the *chi-square test* or *Fisher’s exact test* was used for the comparison between groups of count data. In this study, all measurement data did not conform to the normal distribution, so the non-parametric test method (*Mann-Whitney U test*) was used for the comparison between groups. Binary Logistic regression analysis was used for multivariate analysis. *P*<0.05 indicated that the difference was statistically significant.

## 3 Results

### 3.1 Basic participant characteristics

In this study, a total of 300 questionnaires were distributed, and 293 valid questionnaires were collected, resulting in a valid return rate of 97.7%. There was a relatively balanced distribution of male and female patients, 51.88% vs. 48.12% respectively. The age of the study population was 54.32 ± 9.85 years old. Regarding educational background, 38.23% of the patients had only an elementary school education or below. The majority of the participants were married with a percentage of 89.42%. In terms of economic status, 41.64% of the participants’ households reported a per capita monthly income ranging from ¥3,001 to ¥8,000. For medical expense payment, rural cooperative medical care was mainly adopted, with 178 cases (60.75%). Additionally, 62.46% of the participants had no previous history of cardiovascular diseases. Regarding the type of surgery, 48.50% underwent valve replacement surgery. After the operation, more than half of the patients did not have drainage tubes indwelling, while 43.00% reported experiencing moderate postoperative pain. The duration of the operation in our study population was 240 (190, 290.5) minutes, and the time of CPB was 137 (108, 174.5) minutes. The majority of patients (41.03%) began ambulating for the first time between 4–5 days post-surgery. Furthermore, 61.10% of the patients expressed concerns about the safety of getting out of bed. In terms of postoperative accompaniment, 90.40% of the patients were accompanied by family members. The general characteristics of the participants are summarized in **Table 1**.

**Table 1:** General characteristics of patients after cardiac surgery under CPB (n=293).

Variables	Groups	Frequency	Percentage (%) P50 (P25, P75)
Gender ^a^	Male	152	51.88
	Female	141	48.12
Age (years)^a^	18~44	40	13.65
	45~59	159	54.27
	≥60	94	32.08
Education background ^a^	Primary education or less	112	38.23
	Secondary education	106	36.18
	Senior education	42	14.33
	College or above	33	11.26
Marriage ^a^	Never married	16	5.46
	Married	262	89.42
	Divorced	8	2.73
	Widowed	7	2.39
Household monthly per capita income (RMB)^a^	≤ ¥3000	84	28.67
	¥3001~¥8000	122	41.64
	≥ ¥8001	87	29.69
Payment method ^a^	Private	78	26.62
	Rural cooperative medical insurance	178	60.75
	Employee medical insurance	37	12.63
past history of cardiovascular diseases ^b^	No	183	62.46
	Yes	110	37.54
Operation mode ^b^	CABG	42	14.30
	Valve replacement	142	48.50
	Valve repair	77	26.30
	Other^*^	32	10.90
Number of drainage tubes (root)^b^	0	152	51.90
	1~3	128	43.70
	≥4	13	4.40
Pain level ^b^	None (NRS:0)	0	0
	Mild (NRS:1-3)	106	36.20
	Moderate (NRS:4-6)	126	43.00
	Severe (NRS:7-10)	61	20.80
Operative time ^b^			240 (190, 290.5)
CPB duration ^b^			137 (108, 174.5)
Initial postoperative ambulation time(day) ^b^	≤3	109	37.20
	4~5	121	41.30
	≥6	63	21.50
Safety Concern ^c^	Mild	114	38.90
	Moderate	97	33.10
	Severe	82	28.00
Companionship ^a^	Spouse	164	56.00
	Children or Parents	101	34.40
	Nursing assistants	28	9.60

a demographic factor.

b clinical factor.

c psychological factor.

^*^including: cardiac vascular replacement/repair, resection of mediastinal tumor, and heart transplantation.

### 3.2 The general characteristics of measurements

The total score of kinesiophobia in 293 patients after cardiac surgery under CPB was 37 (34, 42) points, which was at the cut-off value of kinesiophobia. Among them, 134 patients had a score > 37 points, and the incidence of kinesiophobia in this study population was 45.73%. In addition, the findings showed that the overall exercise self-efficacy of the study population was generally at a moderate level, with 195 (66.60%). 98 (33.45%) patients had pain catastrophizing, 168 (57.34%) patients had type D personality traits, and the vast majority of patients (201 patients, 68.60%) had good family functions.

### 3.3 Influencing factors of kinesiophobia in patients after cardiac surgery under CPB

**Figure 1:**
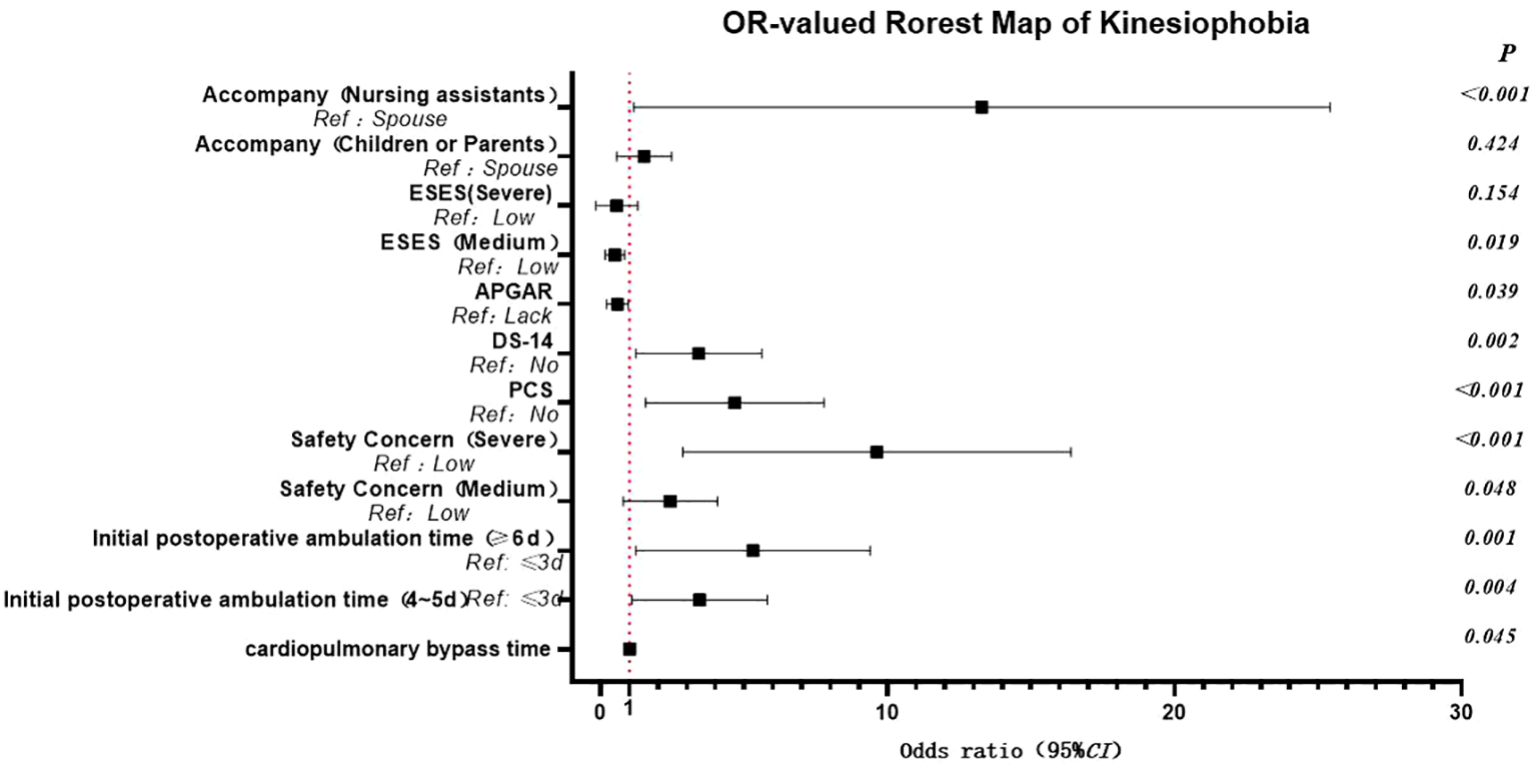
Visualization map of kinesiophobia risk ratio.

The results of the logistic regression analysis identified several risk factors for kinesiophobia in patients following cardiac surgery under CPB. Including having a companion as a nursing assistant, a longer duration of CPB, a delay in the first ambulation after the operation, type D personality, heightened concerns about safety, and pain catastrophizing. Conversely, good family function emerged as a protective factor. Specifically, patients accompanied by nursing assistants exhibited a significantly higher risk of kinesiophobia, increasing by 8.911 times compared to those accompanied by their spouses. Compared to those with a lack of family functioning, the odds of good family functioning for exercise fear decreased by 0.502 times. The prolongation of the CPB duration during the operation was significantly associated with an increased risk of postoperative kinesiophobia. The timing of the first postoperative ambulation also had a substantial impact on kinesiophobia. Patients who initiated ambulation 4–5 days post-surgery had a 2.899 times higher risk compared to those who began ambulating 2–3 days after surgery. For patients who first ambulated 6–7 days post-surgery, the risk escalated to 4.256 times higher. In addition, compared with non-type D personality patients, Type D personality patients had 2.945 times increased risk of kinesiophobia, aligning with previous research (26). The degree of patients’ safety concern was also closely related to kinesiophobia. Patients with moderate concerns had a 2.058 times higher risk, while those with severe concerns faced 7.366 times increase in risk. Pain catastrophizing was an independent risk factor for kinesiophobia, and the risk of patients with pain catastrophizing was 3.987 times higher than that of those without catastrophizing, this view has been confirmed by previous studies (27, 28). Moreover, exercise self-efficacy was found to have a notable influence on kinesiophobia, as shown in **Table 2** and **Figure 1**.

**Table 2:** Binary Logistic regression analysis of kinesiophobia in patients after cardiac surgery under CPB.

Variables	*B*	*S.E*	*Wald χ^2^*	*P*	*Exp (B)*	*95% CI*
CPB duration	0.005	0.003	4.005	0.045	1.005	1.001~1.010
Initial postoperative ambulation time						
≤3 days			13.001	0.002		Ref
4~5 days	1.064	0.373	8.134	0.004	2.899	1.395~6.023
≥6 days	1.448	0.426	11.556	0.001	4.256	1.846~9.808
Safety Concern						
Mild			22.925	<0.001		Ref
Moderate	0.722	0.366	3.898	0.048	2.058	1005~4.215
Severe	1.997	0.418	22.818	<0.001	7.366	3.246~16.712
Companionship						
Spouse			14.985	0.001		Ref
Children or Parents	0.271	0.339	0.638	0.424	1.311	0.675~2.548
Nursing assistants	2.187	0.565	14.964	<0.001	8.911	2.942~26.993
ESES						
Low			5.642	0.060		Ref
Medium	-0.886	0.379	5.471	0.019	0.412	0.196~0.866
High	-0.906	0.635	2.037	0.154	0.404	0.117~1.402
APGAR	-0.690	0.334	4.260	0.039	0.502	0.261~0.966
DS-14	1.080	0.347	9.713	0.002	2.945	1.493~5.807
PCS	1.383	0.360	14.779	<0.001	3.987	1.970~8.070
Constant	-2.761	0.715	14.902	<0.001	0.063	

ESES is the Exercise Self-Efficacy Scale, APGAR is the Family APGAR Index Questionnaire, DS-14 is the Type Personality Scale-14, PCS is the Chinese Version of the Pain Catastrophizing Scale.

## 4 Discussions

This investigation revealed that the incidence of kinesiophobia in patients after cardiac surgery under CPB was as high as 45.73%, which was lower than the incidence in postoperative patients undergoing cardiac surgery through either midline sternotomy or thoracotomy (64.5%) (29). The discrepancy may be attributed to the inclusion of critically ill patients, such as those undergoing coronary artery bypass grafting or aortic dissection, in the open-heart surgery cohort. Previous studies reported that the incidence of kinesiophobia in patients after total knee replacement and thoracolumbar spine fracture surgery was 24.4%-38.2% (30, 31) and 31.88% (32) respectively, significantly lower than that after cardiac surgery under CPB. Such differences might stem from the unique characteristics of cardiac surgery under CPB, including hemodynamic instability, the use of larger doses of vasoactive drugs, prolonged mechanical ventilation, and slower recovery of cardiac function. These factors collectively contribute to increased physical and psychological discomfort in patients, resulting in a higher incidence of kinesiophobia. The findings of this study indicated that the factors influencing kinesiophobia were multifaceted, encompassing socio-demographic, clinical, and psychological factors.

Socio-demographic factors mainly manifested in the impact of the companion on kinesiophobia. Patients accompanied by nursing assistants exhibited a significantly higher risk of kinesiophobia compared to those accompanied by their spouses. This may be attributed to the relatively slow recovery of physical and mental functions following CPB surgery, coupled with lower levels of awareness and acceptance of the condition, leading to increased distress. Nursing assistants were primarily responsible for patients’ daily care rather than providing comprehensive, 24-hour monitoring and companionship, and therefore might not fully understand the patients’ true feelings and needs, which could diminish patients’ psychological sense of security and potentially trigger kinesiophobia.

Clinical factors influencing kinesiophobia included the duration of CPB and the timing of first postoperative ambulation. The duration of CPB might increase kinesiophobia risk through several mechanisms. First, it could lead to reperfusion injury, release of inflammatory mediators, and microthrombi formation, potentially causing complications like pulmonary infections, atrial fibrillation, and neurological dysfunction (33–35). These complications could affect cardiac function and lead to cognitive biases about patients’ abilities, inducing kinesiophobia. Second, longer bypass durations indicated more severe conditions and higher surgical complexity (33), increasing patients’ health concerns and fear of exercise-induced harm. Last, given the prolonged disease course and multiple invasive procedures associated with cardiovascular disease, patients were eager to return to normal life and had heightened needs for rehabilitation. Unmet rehabilitation needs after surgery could heighten insecurity and fear.

Delayed postoperative ambulation was also linked to kinesiophobia. This delay might reflect disease severity, with more severe conditions leading to lower activity confidence and increased fear. Patients with delayed ambulation were also more prone to complications like delirium, pulmonary infections, and muscle atrophy, which could increase perceived danger and induce kinesiophobia. Acute postoperative pain significantly impacted kinesiophobia, as cardiac valve surgery often involves sternotomy and multiple drainage tubes, causing pain that lasts up to 4–6 days. Patients starting activities within 3 days experienced less pain and lower kinesiophobia incidence (36). Finally, insufficient nursing care during the ICU transition period could contribute to kinesiophobia (37). While in the ICU, patients received early rehabilitation education and assistance, reducing kinesiophobia risk. However, those transferred to general wards might face limited rehabilitation guidance, feeling neglected and vulnerable, and fearing re-injury from improper exercise.

Psychological factors included family function, Type D personality, degree of safety concern, and pain catastrophizing. Family function was crucial for patients. It not only provided key resources for adapting to environmental changes but also enhanced patients’ confidence in overcoming the disease. For patients after cardiac surgery under CPB, sternum damage and slow wound healing made them prone to cognitive biases and kinesiophobia. Patients lacking family care and support often showed negative attitudes of avoiding and refusing exercise. Conversely, family companionship and support could help patients better understand the disease and actively cope with postoperative discomfort, thus effectively overcoming kinesiophobia.

The relationship between Type D personality traits and kinesiophobia could be understood from both socio-psychological and biological perspectives. Socio-psychologically, individuals with Type D personality often experience insecurity, anxiety, depression, and pessimism (38), and the accumulation of these emotions leads to increased psychological distress and induced kinesiophobia. Additionally, these patients had weaker social skills, limited disease knowledge, and insufficient understanding of exercise benefits, often restricting activity due to fears that improper exercise might affect recovery. Biologically, Type D personality, as a chronic stressor, impacts stress responses and contributes to coronary heart disease through mechanisms like hormone secretion, inflammation, and vascular endothelial damage, thereby increasing discomfort and leading to kinesiophobia (39).

The degree of patients’ safety concerns was directly proportional to their risk of kinesiophobia, primarily manifesting as fear of falling. This fear might be attributed to postoperative discomforts like fatigue and nausea, which impair physical function and balance, reducing self-control and triggering kinesiophobia. Additionally, the complexity of CPB surgery and potentially severe complications, such as massive bleeding and acute renal failure, necessitate ICU monitoring. The ICU hospitalization experience might lead patients to underestimate their functional abilities and cause them to overly restrict their activities (40).

Pain catastrophizing was an independent risk factor for kinesiophobia because, according to the Fear-Avoidance Model (41), patients equated pain with injury, resulting in intensification of postoperative pain and triggering a psychological stress response that generated fear and avoidance of activities. Xu et al. (28) found that pain catastrophizing had a direct positive effect on the aggravation of kinesiophobia in patients after total knee arthroplasty. Additionally, pain catastrophizing was a key psychological factor in pain perception, and postoperative pain was common, affecting more than 80% of patients, with 20% to 56% experiencing moderate to severe pain (42). Large incisions and prolonged cardiac surgery released inflammatory factors, worsening pain catastrophizing and increasing kinesiophobia risk. Meta-analyses showed that reducing pain catastrophizing and pain levels could effectively reduce kinesiophobia (43).

## 5 Conclusions

This study revealed a high incidence of kinesiophobia in post-cardiac surgery patients under CPB, influenced by complex factors including companionship, family function, bypass duration, time to first ambulation, Type D personality, safety concerns, and pain catastrophizing. To mitigate kinesiophobia and enhance early activity compliance, we recommend: enhancing clinical focus by training healthcare staff on psychological assessment and integrating routine kinesiophobia screening for timely intervention; establishing robust support systems through improved medical insurance to alleviate financial burden and family encouragement for emotional support; implementing the Enhanced Recovery After Cardiac Surgery (ERACS) program, prioritizing multimodal interventions such as optimized pain management, early extubation, and structured activity; and leveraging emerging technologies like cognitive-behavioral therapy, exposure therapy, pain neuroscience education, and virtual reality to deliver individualized rehabilitation plans and overcome resource limitations.

## 6 Strengths and limitations

While research on kinesiophobia in cardiovascular disease has predominantly focused on patients with coronary artery disease and heart failure, few studies have addressed this phenomenon in patients recovering from cardiac surgery. These postoperative patients face a heightened risk of exercise fear due to surgical trauma and wound pain. Therefore, this study investigated the status and characteristics of kinesiophobia specifically in cardiac surgery patients who underwent CPB. Starting from the perspective of the role of stress, this study took the theoretical model of the stress system process as the theoretical basis, combined with the patient-specific indicators of postoperative cardiac surgery under CPB, and clarified the main influencing factors of kinesiophobia in this population.

Nevertheless, there are some limitations in this study. Firstly, this was a cross-sectional study and could not observe the changing trend of patients’ kinesiophobia at different time points. Longitudinal studies should be carried out in the future to explore the development trajectory of kinesiophobia in patients after cardiac surgery under CPB. Secondly, the research participants were only from a single research center, which is a small sample size and has a certain bias. In the future, multi-center and large-sample studies need to be carried out to increase the credibility and stability of the research results. Finally, the questionnaires used in this study relied solely on patient self-reporting, which introduces inherent subjectivity and lacks robust objective data support (e.g., ASA scale, APACHE II score, frailty, high BMI, and low cardiac function). Therefore, incorporating more objective indicators in future research would be valuable to identify more specific and comprehensive influencing factors.

## Data Availability

The original contributions presented in the study are included in the article/**Supplementary Material**. Further inquiries can be directed to the corresponding authors.
