# Supplementary material for: Kinesiophobia and associated factors among patients after cardiac surgery under cardiopulmonary bypass: a cross-sectional study
Source: Front Psychiatry. 2025 Jul 14;16:1584789. doi: 10.3389/fpsyt.2025.1584789 (PMC12301360; doi:10.3389/fpsyt.2025.1584789)
Supplement: Supplementary file 1 [file DataSheet1.pdf]

ID	gender	age	educational level	marital status	household monthly per capita income	payment method of medical expenses
1	1	45	2	2	1	2
2	2	41	2	3	2	2
3	2	61	1	2	2	3
4	1	58	2	2	2	2
5	2	53	1	2	1	2
6	1	52	1	2	1	2
7	2	41	1	2	2	2
8	1	62	2	2	2	1
9	1	42	3	2	1	3
10	2	28	4	2	1	3
11	2	66	2	2	2	3
12	2	45	2	2	1	3
13	1	67	2	2	2	2
14	2	52	1	2	2	2
15	1	55	2	2	1	2
16	2	61	3	2	2	3
17	1	63	3	2	2	1
18	1	50	3	2	3	2
19	1	44	2	2	1	3
20	1	61	1	2	2	2
21	2	65	2	2	2	1
22	2	55	1	2	3	2
23	2	65	1	2	3	1
24	1	67	1	2	1	2
25	1	56	1	3	3	2
26	1	52	1	2	3	2
27	2	59	1	2	2	1
28	2	66	1	2	2	1
29	2	55	2	2	2	2
30	1	42	3	1	1	3
31	1	57	2	2	1	1
32	1	54	1	2	3	2
33	1	66	1	2	3	1
34	2	62	2	2	2	1
35	2	59	2	2	2	3
36	1	58	2	2	3	2

37	2	56	2	2	2	2
38	1	56	2	2	1	2
39	1	58	1	2	1	1
40	1	49	1	2	1	2
41	1	55	1	3	1	1
42	1	58	2	2	1	2
43	2	36	4	1	1	3
44	2	50	1	2	3	1
45	2	43	2	2	1	3
46	1	60	1	2	1	2
47	2	67	2	3	2	2
48	1	56	2	2	2	2
49	1	66	1	2	1	1
50	1	60	2	2	1	1
51	1	50	2	1	1	3
52	2	52	1	2	3	1
53	1	66	1	2	1	1
54	1	46	3	2	1	3
55	1	64	4	2	2	3
56	2	64	2	2	2	3
57	2	40	4	2	1	3
58	2	51	1	2	2	1
59	2	53	1	2	1	2
60	2	51	1	2	1	2
61	1	52	2	2	1	2
62	1	63	4	1	2	2
63	2	65	1	4	2	2
64	2	48	3	2	1	3
65	1	44	2	2	1	3
66	2	55	1	2	1	1
67	2	65	1	2	3	1
68	2	64	2	2	2	2
69	2	34	4	2	1	3
70	2	65	3	2	2	2
71	1	60	2	2	2	2
72	1	67	1	2	2	2
73	1	63	4	2	2	2
74	2	56	1	2	2	1
75	1	64	2	2	3	1
76	2	62	1	2	3	1

77	1	64	1	4	2	2
78	2	69	2	2	2	1
79	1	66	1	2	2	2
80	2	55	1	2	2	2
81	1	56	2	2	3	3
82	1	51	4	2	1	3
83	2	67	1	2	2	1
84	2	64	1	4	3	2
85	1	67	1	2	2	1
86	1	68	1	2	1	2
87	2	50	1	2	3	2
88	1	67	3	2	2	3
89	1	56	1	2	1	2
90	1	46	2	2	1	3
91	1	59	3	2	1	3
92	1	43	3	2	1	3
93	2	54	1	2	1	3
94	1	33	4	2	1	3
95	2	47	1	2	1	2
96	1	53	2	2	1	3
97	2	63	2	2	2	2
98	2	68	1	2	3	1
99	2	58	1	2	2	1
100	1	57	1	2	1	3
101	2	52	1	2	1	2
102	1	57	2	1	3	3
103	2	58	1	2	3	2
104	2	51	1	2	1	3
105	1	62	3	2	2	2
106	2	18	2	1	2	2
107	2	55	3	2	2	2
108	2	53	1	2	3	1
109	2	56	1	2	2	1
110	1	58	2	2	1	3
111	2	28	4	2	1	3
112	1	50	2	2	1	3
113	1	59	2	3	3	2
114	1	59	2	2	3	2
115	1	48	2	2	1	3
116	1	69	1	2	3	1

117	1	58	2	2	1	2
118	2	64	1	2	3	1
119	2	63	2	2	1	2
120	1	48	2	2	3	3
121	1	57	2	2	3	3
122	1	55	2	2	3	2
123	1	41	4	2	1	3
124	2	50	2	3	1	3
125	2	35	3	2	3	2
126	1	32	4	1	1	3
127	1	49	2	2	1	3
128	2	24	4	1	1	3
129	1	46	2	2	1	3
130	1	62	1	2	1	2
131	2	47	2	2	3	3
132	2	55	2	2	2	2
133	1	46	4	2	1	3
134	1	53	2	2	2	3
135	2	59	3	2	3	2
136	1	49	2	2	2	3
137	1	62	1	2	2	2
138	2	56	2	2	3	2
139	1	55	1	2	1	2
140	2	49	2	2	1	3
141	1	68	4	2	2	2
142	1	49	3	2	1	3
143	2	46	3	2	1	3
144	1	44	2	3	1	2
145	2	50	2	2	1	2
146	2	52	2	2	3	2
147	2	57	1	2	2	1
148	2	32	4	2	1	3
149	1	66	3	2	2	2
150	2	61	1	2	3	1
151	2	53	1	2	3	2
152	2	59	1	2	2	1
153	1	47	2	2	1	3
154	1	31	1	2	3	3
155	1	46	2	2	1	2
156	1	47	4	1	1	3

157	1	53	1	2	1	2
158	1	57	2	2	3	3
159	2	31	2	2	1	2
160	1	67	2	2	2	1
161	1	50	1	2	1	3
162	2	52	1	2	3	1
163	1	35	4	2	1	2
164	1	54	2	2	1	2
165	2	60	3	2	3	2
166	2	61	1	2	3	2
167	2	64	1	2	3	1
168	1	55	2	2	1	2
169	1	67	2	2	1	1
170	1	52	2	2	1	1
171	1	31	3	2	1	3
172	2	68	3	2	2	2
173	2	28	4	1	1	3
174	1	67	3	2	2	2
175	1	48	1	2	1	3
176	1	46	1	2	2	2
177	1	50	1	2	3	3
178	1	64	3	2	2	2
179	1	42	4	1	1	3
180	1	56	1	2	2	1
181	2	60	1	2	3	1
182	2	64	2	2	2	1
183	1	61	3	2	3	2
184	1	62	2	2	3	1
185	1	60	3	2	1	3
186	1	59	4	2	3	3
187	2	42	4	2	1	3
188	2	65	1	2	3	1
189	1	65	2	2	3	1
190	2	62	2	2	2	2
191	2	59	2	2	3	1
192	2	52	2	2	2	2
193	2	45	2	2	3	3
194	1	62	1	2	2	2
195	2	34	4	3	1	3
196	1	57	2	2	1	2

197	1	57	1	1	1	3
198	1	56	2	2	2	2
199	2	26	2	2	1	2
200	2	60	2	2	3	1
201	1	36	4	1	1	3
202	1	62	1	2	1	3
203	1	62	1	2	2	1
204	2	40	2	2	1	2
205	2	63	2	2	2	1
206	2	61	2	2	3	1
207	2	56	1	2	3	1
208	2	64	3	2	2	2
209	2	47	4	2	1	3
210	1	70	3	2	2	2
211	2	57	1	2	3	1
212	1	38	4	2	1	3
213	1	58	2	2	3	1
214	2	42	1	2	1	2
215	2	59	1	2	3	1
216	1	67	1	2	1	2
217	2	60	1	4	3	1
218	1	51	2	2	1	2
219	2	49	1	2	3	1
220	2	54	1	2	2	1
221	2	53	1	2	3	3
222	2	58	3	2	2	2
223	1	50	4	2	1	3
224	2	57	1	2	1	1
225	1	59	2	2	1	2
226	2	46	2	2	3	1
227	1	61	3	2	2	2
228	2	49	3	2	3	2
229	2	47	1	2	1	1
230	2	51	1	2	1	2
231	2	59	1	2	3	1
232	1	64	1	2	1	2
233	1	64	1	2	1	2
234	2	53	1	2	3	1
235	1	60	3	2	2	2
236	1	59	4	2	1	8

237	2	50	1	2	1	2
238	2	59	1	2	3	1
239	1	58	3	2	1	2
240	2	59	3	4	3	1
241	2	57	2	2	1	2
242	2	54	1	2	1	2
243	2	62	1	2	3	1
244	1	56	2	2	1	3
245	1	46	3	2	1	2
246	1	50	1	2	1	2
247	2	46	1	2	3	3
248	1	59	3	1	3	1
249	2	28	2	2	3	2
250	1	57	2	2	1	2
251	2	48	2	2	1	2
252	1	36	2	2	1	2
253	1	66	1	2	3	1
254	1	52	4	2	1	3
255	2	65	2	2	1	2
256	2	52	2	2	3	1
257	2	54	1	2	1	2
258	2	58	1	2	1	2
259	1	58	3	2	1	2
260	1	52	1	2	3	1
261	1	55	3	1	1	3
262	2	65	1	4	2	1
263	2	48	3	2	1	3
264	2	56	2	2	1	2
265	1	57	1	2	1	3
266	2	64	1	2	2	1
267	2	65	1	2	3	1
268	1	52	1	2	3	1
269	1	69	2	2	3	1
270	2	65	1	2	1	2
271	2	51	1	2	1	1
272	2	38	2	2	3	1
273	1	68	3	2	3	1
274	2	61	1	2	3	1
275	2	53	2	2	2	2
276	2	50	2	2	1	3

277	1	41	3	2	1	3
278	2	57	1	2	3	1
279	1	64	1	2	3	1
280	1	59	2	2	1	2
281	1	67	2	2	3	1
282	1	34	3	2	1	3
283	2	28	4	2	1	3
284	2	52	2	2	2	1
285	1	67	4	2	2	2
286	1	46	2	1	1	3
287	1	49	2	2	1	3
288	1	59	4	2	1	3
289	1	69	2	4	2	1
290	2	46	3	2	1	2
291	1	52	2	2	1	2
292	1	61	4	2	2	2
293	2	58	2	2	2	1

past history of cardiovascular disease	past history of cardiovascular disease	operation type	operation time	cardiopulmonary bypass time
1	2	2	270	144
2	1	2	250	135
3	2	2	320	235
2	2	2	165	80
1	2	3	260	67
3	2	2	205	126
2	2	2	240	120
2	2	1	480	326
1	2	4	375	254
1	2	3	190	80
2	2	5	165	76
1	1	2	180	68
2	2	2	210	160
2	2	6	160	117
2	2	2	235	149
2	2	5	370	183
2	2	2	345	226
2	2	1	320	191
1	2	2	265	140
2	2	1	275	141
2	1	2	230	140
2	2	3	205	116
2	2	3	259	146
2	2	1	300	181
2	2	1	305	122
3	2	2	220	132
2	2	3	162	106
2	1	2	240	130
3	2	2	220	100
3	1	2	225	109
2	2	2	145	88
2	2	3	235	143
2	1	2	322	124
3	2	3	280	130
2	1	2	235	123
2	2	2	295	141

2	1	2	200	161
2	1	2	315	179
2	1	2	145	77
3	2	3	270	163
2	2	2	257	129
2	1	2	420	346
1	2	3	150	87
2	1	3	210	150
1	2	2	150	103
3	2	5	170	87
2	2	2	210	113
3	2	1	305	260
3	2	1	323	143
2	1	3	190	144
1	2	2	370	154
2	2	2	240	124
2	2	1	355	129
1	1	5	270	140
2	2	2	280	206
2	1	4	180	50
1	1	3	135	99
2	2	2	295	181
1	2	2	240	115
1	2	2	260	140
3	2	3	200	145
2	2	2	300	260
2	2	1	325	220
1	2	4	95	30
1	1	3	200	125
2	1	2	235	134
2	1	2	225	146
2	1	1	375	165
1	1	3	110	35
2	2	1	235	200
3	2	1	440	163
2	2	1	280	117
2	2	5	315	177
3	1	3	205	155
3	2	3	240	64
2	1	2	195	131

2	1	2	240	153
2	2	2	295	195
2	2	1	360	180
3	1	2	315	207
2	1	2	225	148
1	2	3	270	149
2	2	2	260	127
2	1	2	130	91
3	2	4	190	69
2	2	5	291	183
2	2	2	275	174
2	1	2	240	139
2	2	2	320	165
1	2	2	220	109
1	2	1	285	135
1	2	5	245	130
2	2	3	140	74
1	1	1	375	237
1	1	4	100	60
1	2	2	240	178
2	2	1	385	231
3	1	2	220	130
2	2	2	215	132
2	1	1	323	190
1	1	2	300	164
2	2	3	275	198
2	1	2	285	167
3	2	3	165	80
3	2	3	200	141
3	2	3	240	133
2	2	1	345	191
2	2	2	140	82
2	2	4	145	66
2	2	3	275	201
1	2	3	215	138
1	2	1	325	209
2	2	1	90	30
2	1	2	215	139
1	2	4	125	60
2	2	1	290	200

2	2	1	230	165
3	1	2	220	125
2	2	2	240	111
2	2	4	125	75
2	1	2	260	163
3	2	2	245	150
1	2	3	270	164
1	2	5	600	243
2	2	3	125	45
1	2	5	400	249
1	1	1	425	239
1	2	1	475	360
1	2	3	196	128
2	2	2	240	160
2	2	3	120	65
2	1	2	240	171
1	2	1	185	131
2	2	5	280	205
2	1	2	120	70
2	1	2	180	125
2	2	2	125	65
2	2	1	275	172
2	2	3	180	76
1	1	2	240	150
2	2	4	180	100
1	2	2	225	127
1	2	5	160	83
1	2	5	300	186
1	1	2	240	150
2	2	2	130	79
2	2	2	155	66
1	2	3	180	84
2	1	1	320	145
2	2	2	220	180
2	1	5	370	280
2	2	2	225	114
1	1	2	180	119
2	2	3	148	68
1	2	1	330	165
3	2	3	460	364

1	2	2	225	98
2	2	2	180	91
1	1	3	125	69
2	2	1	336	171
1	2	5	265	160
2	2	2	215	146
1	2	6	270	107
2	2	2	122	70
2	2	1	40	100
2	1	2	195	129
2	2	2	240	130
2	2	2	320	215
2	2	2	240	116
1	1	1	335	133
1	2	3	205	120
2	1	2	180	84
1	2	3	200	108
2	2	1	230	125
1	1	2	200	106
2	2	3	180	95
2	1	3	180	101
2	2	2	220	86
1	1	3	510	290
2	1	2	225	140
2	2	2	360	176
2	1	1	300	154
2	2	5	225	106
2	2	5	265	185
1	2	3	325	254
2	2	2	270	180
1	2	4	240	162
2	1	3	180	104
3	2	2	180	99
2	1	4	180	88
2	1	2	259	166
2	2	2	195	130
2	2	2	260	192
2	1	1	330	199
1	2	2	205	122
2	1	2	225	121

2	2	1	240	161
2	2	5	350	215
1	1	3	180	85
2	2	2	240	139
1	1	3	180	123
2	1	5	340	210
2	2	2	325	251
1	1	3	150	34
2	1	2	240	100
2	2	2	335	225
2	2	2	290	240
2	2	2	225	168
1	2	2	305	164
2	1	3	210	126
2	2	2	180	118
1	2	1	310	196
2	1	3	240	101
1	1	2	300	175
2	2	2	200	131
2	2	3	215	135
2	2	3	215	157
1	2	2	240	180
2	1	3	130	65
2	1	2	310	207
2	1	3	185	99
2	2	2	200	83
1	2	3	180	120
2	2	2	240	148
2	2	2	280	181
2	1	3	280	208
2	2	1	300	175
2	1	3	270	128
1	2	2	240	147
1	2	2	150	92
2	2	2	180	109
2	1	2	217	66
2	2	2	180	118
2	2	3	270	172
2	2	3	240	100
1	1	2	190	141

1	1	2	300	125
3	1	2	240	159
1	2	3	245	108
2	2	2	180	108
2	2	3	225	119
2	2	3	240	113
2	1	3	240	131
2	2	1	240	160
3	2	1	350	245
3	2	2	230	122
2	2	2	180	85
2	1	2	300	117
2	2	3	210	137
2	1	5	420	293
1	2	2	180	126
1	2	2	180	122
3	1	2	240	120
1	2	1	290	182
2	1	3	195	76
3	2	3	180	109
2	2	2	240	121
2	1	1	230	137
1	1	3	240	145
2	1	2	330	185
1	2	2	280	156
3	2	2	325	220
2	2	3	95	30
1	2	2	420	151
2	2	3	420	254
2	1	2	255	166
3	2	2	180	95
3	2	2	440	300
1	2	2	195	135
3	2	3	195	130
1	1	3	260	180
3	2	2	210	155
3	2	3	320	228
3	2	2	195	115
2	2	2	240	165
2	2	3	220	120

2	2	3	190	115
1	2	2	180	100
1	2	3	210	115
3	2	3	246	160
1	2	2	260	160
2	2	2	240	180
2	1	3	95	40
2	1	3	120	55
2	2	2	480	350
2	2	2	435	360
2	2	2	280	162
2	2	3	190	134
2	2	3	430	251
2	2	2	180	95
2	2	2	240	165
2	2	3	265	198
2	2	2	300	210

severity of pain	tinitial postoperative ambulation time	safety concern	companion	Type D-1	Type D-2	Type D-3
4	3	3	1	2	0	0
3	3	2	2	1	0	0
4	6	3	2	4	3	2
2	3	1	2	1	1	1
5	4	3	1	4	4	4
3	3	2	3	4	0	0
4	5	3	1	1	0	2
7	6	2	2	2	0	4
3	2	3	1	2	1	1
2	2	3	1	2	2	2
4	3	2	2	4	1	1
2	3	2	1	1	1	1
3	4	2	2	3	3	1
4	3	2	1	3	1	3
4	3	2	3	4	0	2
8	9	3	2	2	1	1
4	3	2	2	3	1	1
2	4	1	1	3	0	1
4	4	2	1	4	0	4
5	8	1	3	0	1	3
2	3	1	2	3	0	1
3	4	2	2	3	1	2
3	3	1	2	2	0	1
5	3	3	2	1	1	1
5	3	2	1	1	2	1
6	3	1	1	1	0	1
8	4	3	2	1	3	2
5	3	2	2	1	3	1
6	5	2	2	1	1	1
2	5	1	3	4	0	1
5	6	2	2	1	0	1
8	7	2	1	3	2	1
3	7	1	2	1	2	1
8	5	3	2	1	2	2
5	2	1	2	2	1	1
7	4	2	2	4	0	0

0	3	3	2	4	0	4
7	4	2	3	2	1	1
6	4	2	1	3	1	1
6	3	2	1	2	2	2
3	4	2	2	1	2	1
4	6	2	1	0	1	2
7	7	1	3	4	1	1
7	7	3	3	2	1	2
5	3	1	3	2	2	3
5	4	1	2	2	0	1
0	11	2	1	4	2	3
7	4	3	3	1	2	1
7	2	1	2	1	1	2
3	3	1	2	1	2	2
5	6	1	3	0	2	2
2	5	3	1	4	3	4
3	3	2	2	1	1	1
6	3	2	3	2	0	4
5	3	1	2	1	0	1
3	2	2	2	4	0	4
3	4	1	3	1	0	2
4	4	2	1	2	1	1
5	3	2	1	2	0	2
5	11	2	1	2	1	1
6	7	2	3	1	2	1
4	6	1	1	2	0	1
6	6	1	1	3	2	1
6	3	1	1	3	2	2
8	4	2	1	2	2	2
7	4	2	1	1	1	2
8	4	2	2	1	1	2
5	3	1	2	3	0	2
5	3	3	1	2	2	2
3	3	3	2	2	0	2
7	4	3	2	1	2	1
6	4	2	2	1	2	2
3	3	1	2	3	1	1
3	3	1	1	1	1	1
2	5	1	3	0	0	0
3	3	1	2	1	1	1

6	3	1	1	1	1	1
5	5	1	2	1	1	1
6	5	3	2	1	1	1
8	4	3	1	4	1	1
5	4	1	1	4	1	1
2	3	2	1	2	1	2
6	3	3	2	3	2	1
7	6	1	1	2	1	1
6	5	3	2	1	2	1
7	5	1	2	1	1	1
7	4	3	1	1	2	1
7	4	2	2	4	1	1
7	4	2	1	1	1	1
6	4	2	1	2	0	0
4	3	1	1	2	0	1
7	3	2	1	3	1	2
6	3	1	1	3	1	2
3	3	1	1	3	0	4
5	3	3	1	2	1	1
5	2	3	1	4	1	1
4	6	1	2	1	1	1
6	4	3	2	1	2	1
6	3	3	1	1	2	1
6	6	1	1	2	0	1
6	6	1	1	3	1	1
3	5	1	1	3	0	1
6	6	1	1	3	2	1
6	4	1	1	1	1	1
1	2	1	2	2	0	0
3	3	2	1	3	2	1
7	4	3	1	3	1	2
6	3	2	1	1	3	1
6	3	1	3	1	2	0
4	4	1	1	2	0	1
8	4	3	1	2	3	2
6	5	2	1	3	0	1
3	2	1	1	3	2	1
5	7	1	1	2	0	2
6	2	2	1	3	1	2
7	5	3	2	1	2	0

4	5	1	1	3	0	1
7	3	3	2	1	3	1
6	2	3	2	2	2	1
4	7	2	1	3	0	1
5	3	1	1	3	0	3
6	3	3	1	1	2	1
2	7	1	3	4	0	3
7	7	1	2	3	0	3
5	2	2	1	2	2	2
6	3	3	3	3	1	2
6	5	2	1	3	0	2
3	7	1	1	4	0	1
6	3	1	1	3	0	1
3	2	3	2	1	1	0
8	3	1	1	2	1	3
0	4	1	1	1	0	1
6	5	3	3	3	2	3
3	3	2	1	2	2	1
5	3	2	1	3	1	0
3	7	2	1	3	0	3
8	6	3	2	2	0	1
7	6	3	1	2	1	1
1	7	1	1	4	0	0
6	5	3	1	3	0	1
3	4	2	2	3	1	3
6	3	2	1	2	1	1
4	3	1	1	3	1	2
6	6	2	2	3	2	3
1	6	2	1	3	1	1
4	2	1	1	1	1	1
5	3	1	1	3	0	2
8	3	1	1	4	0	3
8	7	1	2	3	0	2
5	9	3	2	1	2	1
6	4	3	1	1	1	1
5	5	1	1	3	1	1
5	3	1	1	4	0	1
9	8	3	1	4	0	3
6	8	3	1	2	0	2
4	4	1	3	1	0	1

3	5	2	1	3	0	1
3	5	1	1	2	0	2
6	4	1	1	3	2	2
2	5	1	2	3	0	1
2	4	2	1	4	0	3
7	7	3	1	3	1	1
2	4	2	1	3	1	3
8	7	1	1	2	0	1
3	3	1	2	3	0	1
7	3	2	2	3	2	2
1	2	3	2	2	1	0
1	2	3	1	3	3	2
5	4	3	2	3	0	3
8	5	3	1	1	2	1
7	2	1	1	4	0	2
3	4	1	3	2	0	1
6	5	1	3	4	0	3
7	4	1	2	3	0	1
2	4	1	1	4	0	1
5	4	1	1	1	0	1
2	3	1	1	3	0	2
6	4	1	2	3	3	2
8	3	3	3	2	2	2
2	7	3	1	1	3	1
7	6	2	2	3	1	3
2	14	2	2	1	2	1
5	5	2	2	1	0	1
9	5	2	2	2	1	1
2	5	1	2	4	2	3
7	4	3	1	4	2	3
8	4	3	1	4	0	3
5	6	3	2	3	2	3
2	6	1	2	4	0	3
5	3	3	2	3	1	1
3	4	3	1	1	2	1
3	3	2	1	1	3	1
5	3	2	1	1	2	1
4	6	2	2	1	2	1
3	5	2	2	1	0	3
6	4	2	1	3	1	3

7	4	1	1	1	0	1
3	6	1	1	1	0	1
6	4	3	1	1	3	1
3	8	3	2	3	1	1
1	3	1	3	3	1	3
2	4	1	2	3	1	2
7	3	1	2	1	3	0
2	2	2	1	3	3	1
3	5	2	2	1	2	1
3	5	3	2	2	2	0
6	4	3	1	3	2	2
5	7	3	2	3	3	1
4	7	3	1	3	1	2
1	3	2	2	3	1	1
4	4	1	1	2	1	1
3	3	3	3	3	1	1
7	4	2	1	1	3	1
2	4	2	1	2	3	1
5	3	3	1	1	3	1
5	3	3	2	2	3	1
2	5	2	1	2	3	1
4	6	1	1	1	3	1
8	6	3	1	1	3	2
6	4	3	1	2	3	1
3	6	2	1	3	3	3
7	5	1	1	3	3	2
7	3	1	1	4	0	2
6	4	3	2	2	3	1
4	4	2	2	3	2	2
2	5	1	1	3	3	1
3	4	1	2	3	1	1
5	5	1	1	2	3	2
7	5	1	1	1	3	1
2	5	2	1	3	2	2
7	4	3	2	2	3	0
5	6	2	2	3	1	2
6	4	2	2	1	3	1
4	5	1	1	2	2	1
3	4	2	2	2	0	0
3	6	2	2	3	2	1

7	6	1	1	2	3	1
6	6	2	2	1	2	1
4	3	2	2	3	1	2
1	2	2	1	1	1	1
3	3	2	3	3	2	2
3	3	2	1	2	3	1
6	4	3	2	1	3	1
6	4	3	1	1	3	1
6	3	2	3	2	0	3
6	4	3	1	1	1	1
8	5	1	3	2	3	3
4	7	2	2	1	2	1
1	6	1	1	4	3	3
3	3	1	1	3	1	1
2	4	1	1	3	3	1
1	7	2	1	2	0	1
7	4	3	2	1	3	1
3	4	2	1	2	1	1
2	3	1	2	3	3	2
5	4	2	1	2	1	1
2	4	1	1	1	2	1
2	4	1	1	3	3	3
2	5	1	1	1	1	1
6	3	3	1	1	2	1
7	4	3	1	2	3	1
4	6	1	2	1	3	1
4	3	1	1	3	1	3
7	3	3	1	3	1	2
6	4	3	1	1	3	1
5	6	2	1	2	3	1
5	4	1	1	1	3	1
3	4	2	1	1	1	1
4	3	3	1	1	2	1
1	2	1	1	1	2	1
5	4	2	1	1	3	1
4	6	3	1	1	3	1
3	6	3	2	1	2	1
7	3	3	1	1	3	1
2	2	1	1	3	1	3
2	5	1	1	3	1	3

3	3	1	1	3	1	3
5	2	3	2	1	3	1
2	4	2	2	1	1	1
2	6	1	1	2	1	2
6	3	3	2	1	3	1
2	2	1	1	3	1	3
2	3	1	1	3	1	2
2	2	1	2	3	1	3
7	4	3	1	2	3	1
2	4	2	1	3	1	3
6	5	1	3	3	3	2
6	4	2	1	2	2	1
3	5	1	2	2	0	1
1	3	1	1	3	1	1
5	3	2	1	3	3	1
3	3	1	2	3	1	3
6	4	2	1	1	3	1

Type D-4	Type D-5	Type D-6	Type D-7	Type D-8	Type D-9	Type D-10	Type D-11
0	2	1	0	1	0	0	0
3	1	0	3	0	1	0	0
2	2	1	2	2	2	1	1
3	2	1	3	3	3	2	2
4	1	4	4	4	4	4	4
1	1	2	0	0	1	0	0
0	0	0	0	0	0	0	0
1	0	2	4	1	0	1	0
2	3	2	1	1	2	1	1
2	2	2	2	2	2	3	2
2	2	1	1	2	1	1	1
2	2	3	1	3	1	1	2
3	3	3	1	2	1	3	3
2	2	1	1	2	1	1	2
0	0	0	0	0	0	0	0
1	1	1	0	0	0	0	0
1	2	1	2	1	1	1	4
1	3	0	0	1	0	0	1
2	3	4	1	4	0	0	4
1	2	1	1	1	1	1	2
2	1	1	1	2	2	1	2
1	2	1	1	2	2	0	1
2	1	2	2	3	2	1	2
2	3	2	2	3	2	1	2
2	4	2	1	2	2	3	2
0	2	2	1	2	1	1	2
3	3	2	2	3	3	1	2
2	3	2	1	2	2	1	2
2	2	2	1	2	2	1	2
0	0	0	0	0	0	0	0
1	3	2	2	2	2	1	2
1	1	1	0	0	0	0	0
2	2	2	2	2	2	1	1
3	2	2	2	2	2	2	2
2	2	3	1	2	2	3	2
0	0	0	0	0	0	0	0

0	0	0	0	0	0	0	0
2	2	2	1	2	1	1	2
1	2	2	2	2	1	1	2
2	3	2	1	2	2	1	2
1	1	2	2	2	1	2	1
1	1	1	0	3	0	1	1
0	1	1	1	1	1	1	1
2	1	2	1	2	2	1	2
2	2	1	1	1	2	1	2
3	3	1	0	0	1	0	0
1	0	0	0	0	0	1	0
2	3	2	2	2	1	2	2
2	1	3	2	2	1	1	2
2	1	2	2	2	1	2	2
2	1	2	2	2	1	1	0
2	4	0	1	2	3	2	1
1	1	1	2	2	1	2	2
4	4	4	0	4	4	4	4
2	2	2	1	2	1	2	1
0	1	0	0	0	0	0	0
0	0	0	0	0	0	0	0
2	1	2	1	2	1	0	1
0	0	1	1	1	0	0	0
2	2	2	3	2	2	2	2
0	1	3	0	2	1	0	0
1	2	2	1	2	1	1	1
2	3	2	2	3	2	1	2
2	3	1	2	3	2	1	2
2	2	2	2	2	2	2	2
2	2	2	1	3	2	2	1
2	2	2	3	2	2	2	1
2	3	1	1	2	2	2	1
1	2	1	2	2	2	1	2
0	0	0	0	0	0	0	0
2	3	2	2	2	2	2	1
2	1	1	1	2	2	1	2
2	3	2	1	2	1	1	1
2	3	2	1	1	1	2	2
0	0	0	0	0	0	0	0
2	2	2	1	2	1	2	2

2	2	2	1	2	2	1	2
2	1	1	1	1	1	1	1
2	3	2	2	2	2	1	2
1	2	2	2	2	2	1	1
1	1	0	1	2	1	1	1
1	2	1	2	1	1	1	2
2	3	2	2	2	2	1	2
2	2	2	2	2	1	1	2
2	2	3	2	3	2	2	1
1	2	2	2	2	1	2	1
2	3	2	2	2	2	1	1
2	2	1	1	1	3	1	2
3	3	3	3	2	2	3	2
1	0	0	0	1	1	0	2
1	3	2	1	2	1	1	1
1	3	2	1	2	1	0	1
2	3	2	2	1	1	1	1
2	1	1	1	2	1	3	2
1	2	2	1	2	1	1	2
2	2	2	1	2	1	1	1
2	1	2	1	2	1	1	1
2	2	3	1	2	2	2	1
2	3	3	2	2	2	1	1
1	0	1	1	1	1	1	2
2	1	3	2	2	2	2	1
2	2	2	1	2	1	1	2
3	4	2	2	2	2	2	2
2	3	2	2	2	1	2	2
1	0	2	0	1	0	1	3
1	2	2	1	1	0	0	0
2	1	1	1	1	1	1	1
2	2	2	2	2	1	2	1
1	1	2	2	2	1	2	1
2	1	2	1	2	1	2	0
3	4	1	2	1	3	1	1
2	1	2	1	2	1	1	1
1	2	2	1	1	1	1	2
1	0	0	1	1	1	0	0
1	2	0	2	2	1	1	1
2	3	3	2	3	1	1	1

1	1	2	3	2	2	2	2
3	3	2	3	1	3	2	1
2	3	3	3	2	1	2	2
4	2	1	1	1	1	0	1
0	1	1	1	1	1	1	1
3	3	3	3	3	1	2	1
1	1	1	1	1	1	0	0
3	3	0	2	0	3	0	1
2	2	1	2	0	0	0	0
2	1	1	1	1	1	0	1
1	2	2	1	1	1	1	1
0	0	0	4	0	1	0	4
2	1	2	1	1	1	2	1
2	3	2	2	2	1	2	1
1	2	1	3	1	2	1	2
2	2	2	2	1	1	2	1
3	3	2	3	3	3	3	3
2	3	2	0	3	1	0	2
2	2	2	2	2	1	2	2
1	1	1	3	1	0	1	0
1	2	2	1	1	1	1	2
2	3	3	3	2	2	1	2
0	0	0	1	0	0	0	0
1	2	2	1	2	1	1	1
1	1	1	1	1	1	1	1
2	3	2	2	2	2	1	1
1	1	3	1	1	4	2	2
1	2	1	0	0	2	0	1
2	1	1	2	2	1	1	1
0	1	2	1	2	1	1	1
2	2	1	1	1	1	1	1
1	1	2	1	2	1	1	1
1	1	2	2	1	2	1	2
2	3	3	3	2	2	1	2
2	1	2	1	1	1	1	1
1	3	1	0	1	1	1	1
0	3	0	0	0	0	0	0
0	1	1	1	1	1	1	1
2	1	2	2	2	1	1	2
0	1	2	0	2	0	1	2

0	1	1	1	1	1	1	1
1	1	2	1	1	1	2	2
1	3	2	2	2	2	2	1
1	2	1	2	1	1	1	1
0	1	0	1	1	1	1	1
1	2	2	2	2	1	1	1
1	1	1	0	1	0	0	0
1	4	1	2	1	1	1	1
0	1	2	1	1	0	1	1
2	2	1	2	1	1	1	1
0	2	1	1	2	1	1	1
3	2	2	1	1	2	1	1
1	2	1	1	1	1	0	1
3	2	3	2	2	2	2	1
0	1	0	1	1	1	1	1
1	2	1	1	1	1	0	1
1	0	0	0	0	0	0	0
1	2	1	1	1	1	1	1
0	1	1	1	1	1	1	1
2	2	2	2	1	1	1	1
1	1	1	1	1	1	1	1
2	3	1	2	1	3	0	0
1	1	1	3	1	1	1	2
2	1	2	2	2	2	1	2
1	2	2	2	1	1	1	1
3	2	2	3	1	3	2	2
2	1	2	2	2	1	2	1
1	1	2	2	1	1	2	1
1	2	1	1	1	1	1	2
2	3	1	2	2	1	1	1
1	1	1	1	2	2	2	1
2	3	2	2	1	3	2	2
0	2	0	1	1	1	1	0
3	3	1	2	1	3	1	2
2	1	2	2	2	2	1	1
2	3	2	2	2	1	2	1
2	2	2	2	2	2	1	2
1	2	2	2	2	1	1	2
1	1	0	1	0	1	1	3
0	2	1	2	1	1	1	1

1	2	2	2	2	2	3	2
2	1	1	4	1	2	2	1
2	2	2	2	2	2	2	2
2	2	2	2	1	1	2	1
2	2	1	2	1	2	1	2
1	4	1	2	1	1	1	1
1	2	1	2	1	1	1	2
1	3	2	2	1	1	2	1
2	1	2	2	1	2	1	2
2	2	2	2	1	2	2	1
1	2	2	2	1	1	1	1
1	2	1	1	1	1	1	1
1	2	1	1	1	1	1	0
1	2	1	1	2	1	1	1
1	2	1	1	2	1	0	1
2	2	1	1	2	1	1	1
1	2	2	2	2	1	1	1
3	2	2	2	2	3	2	2
2	3	2	2	1	2	2	1
2	2	2	2	2	2	2	2
2	1	2	2	2	1	2	1
1	3	2	2	2	1	2	2
2	1	2	2	2	1	0	1
2	3	2	2	2	2	1	1
1	2	2	1	1	1	1	1
3	3	2	1	2	1	1	2
0	0	0	0	0	0	0	2
2	2	2	2	2	2	1	2
2	3	1	1	1	2	0	1
1	1	1	1	1	1	1	1
1	1	2	1	1	1	1	1
2	2	1	1	1	2	1	1
2	2	2	2	2	1	2	2
1	2	1	1	1	1	1	0
2	2	2	2	2	2	1	2
1	2	1	1	1	1	1	1
2	2	2	2	2	2	1	2
2	2	2	2	2	1	1	2
0	0	2	0	1	0	0	1
1	2	3	1	1	1	1	2

2	2	2	2	1	2	1	1
1	1	2	1	1	1	1	2
1	3	2	2	1	1	1	1
1	2	1	2	2	1	1	1
2	2	1	2	2	2	1	1
2	2	1	3	1	2	1	1
2	3	2	2	2	2	2	2
2	3	2	2	2	2	2	2
1	3	1	2	2	0	1	1
2	2	1	2	1	2	1	2
3	3	1	2	1	1	1	1
2	2	1	2	2	2	3	1
2	3	1	2	1	2	1	1
2	2	2	2	1	1	1	1
1	3	1	1	1	1	1	1
1	1	1	2	1	1	1	1
2	3	2	3	2	2	2	2
2	2	4	0	1	2	1	1
1	0	2	2	1	1	1	1
1	2	2	1	1	1	1	0
0	1	2	2	2	1	1	1
2	1	1	2	0	1	1	1
1	2	1	1	1	1	1	1
2	3	2	2	2	2	2	1
2	3	2	2	2	2	1	2
1	2	2	2	2	2	3	2
0	1	1	0	0	1	1	1
1	2	1	2	1	2	1	1
2	2	3	2	2	2	2	2
2	4	3	2	2	2	2	2
1	2	2	2	2	2	2	1
1	2	3	2	2	1	1	1
1	3	2	2	2	1	2	1
1	1	2	2	1	1	1	1
2	2	2	2	2	2	2	2
1	2	2	2	2	1	2	2
1	2	1	2	2	1	1	2
2	2	2	3	2	2	2	2
1	0	1	1	1	1	0	1
1	1	1	1	1	1	1	1

1	2	1	1	1	1	1	1
2	2	3	2	2	1	3	2
1	2	2	2	2	1	1	1
1	2	1	2	2	1	1	1
2	3	2	3	3	2	2	2
1	2	1	1	1	1	0	1
1	2	1	1	1	1	1	1
0	1	1	1	1	1	1	1
2	3	2	2	2	2	1	2
0	3	1	1	0	0	1	1
0	2	1	1	1	0	0	1
1	3	1	2	2	1	2	2
2	1	2	1	2	2	2	2
1	1	1	1	1	1	1	1
1	2	1	1	1	1	1	1
0	1	1	1	1	1	1	1
2	2	2	2	2	2	2	2

Type D-12	Type D-13	Type D-14	TSK-1	TSK-2	TSK-3	TSK-4	TSK-5
2	1	1	3	1	1	4	1
0	0	0	3	2	1	3	4
2	2	2	3	3	1	2	2
1	3	2	3	3	1	3	1
4	3	3	4	4	4	4	2
0	0	2	3	2	1	3	3
0	0	0	3	1	1	3	1
2	1	3	3	2	2	3	1
2	2	1	3	2	1	1	1
2	2	2	3	2	1	4	1
2	2	2	3	1	1	2	2
2	1	2	3	3	2	3	2
3	1	1	3	2	2	3	2
2	1	1	1	2	2	4	1
0	0	0	3	2	3	3	1
1	1	1	1	1	1	3	1
2	1	1	3	3	4	2	2
1	0	0	2	1	2	4	3
2	3	3	4	2	1	3	2
2	1	1	2	2	2	3	1
1	1	1	2	2	2	3	1
2	1	1	3	3	2	3	2
2	1	2	3	1	2	3	1
2	2	3	3	2	2	3	1
1	1	2	2	2	1	3	1
1	1	1	2	2	1	2	1
3	2	2	4	2	3	3	1
2	1	2	3	3	3	3	1
2	1	2	3	4	2	2	1
0	0	0	3	3	2	3	1
1	1	2	2	4	3	4	1
3	0	2	1	1	1	3	1
1	1	1	2	2	3	3	2
3	3	2	4	2	3	3	1
2	1	2	2	2	1	4	1
1	0	0	1	1	1	2	2

0	0	0	2	2	3	3	1
2	1	1	2	2	4	3	2
2	1	2	2	2	2	4	1
3	2	2	1	3	2	3	1
2	1	2	2	2	3	3	1
1	0	3	2	3	2	2	2
1	1	1	1	1	1	3	1
1	1	2	4	4	2	2	1
1	2	1	2	3	3	3	2
0	0	0	3	2	1	4	1
3	1	0	4	4	3	2	2
1	2	2	4	3	3	3	2
2	1	2	2	3	2	4	1
2	1	2	2	2	1	3	1
2	2	2	1	2	3	3	2
3	2	2	3	3	2	4	1
2	2	1	2	2	2	3	1
4	4	4	3	4	1	4	4
2	1	2	2	2	1	3	1
0	0	0	1	1	1	1	1
0	0	0	1	2	1	2	1
2	2	1	2	1	2	3	1
1	1	1	2	2	2	3	2
2	2	3	3	2	2	4	1
2	0	3	2	1	2	4	1
2	2	2	2	2	2	4	1
2	1	2	4	2	2	3	2
3	2	1	1	2	2	3	2
2	2	2	3	3	2	2	1
2	2	2	3	3	2	3	1
2	2	2	3	3	2	3	1
1	1	2	2	3	1	3	1
3	1	2	3	1	2	4	1
0	0	0	3	3	2	3	2
3	2	2	4	2	2	3	1
2	1	2	2	2	2	4	1
2	2	1	1	2	2	3	1
1	1	2	1	1	2	3	1
0	0	0	3	3	2	2	1
2	1	2	2	2	2	4	1

1	1	2	2	2	3	2	1
1	1	1	2	2	2	2	1
3	1	2	2	3	2	3	1
3	2	2	2	2	1	4	1
1	1	2	1	2	2	4	1
1	2	2	1	3	2	2	2
3	2	1	4	1	2	3	2
0	0	0	2	2	2	3	1
2	2	2	3	3	2	3	1
3	1	2	3	4	2	2	1
3	1	2	4	3	3	3	1
3	2	1	3	2	2	3	2
3	2	2	4	3	3	2	1
2	1	1	2	1	2	3	2
2	1	2	2	2	2	4	1
2	1	2	3	4	2	3	1
3	2	2	2	3	2	4	1
1	3	1	2	3	1	4	1
3	2	2	2	3	2	3	1
2	1	2	4	2	2	3	1
1	1	2	2	2	2	3	1
3	2	2	3	3	2	3	1
3	2	3	3	3	3	2	2
2	1	1	2	2	2	3	2
3	3	2	2	1	2	4	1
1	2	1	1	1	2	4	1
3	2	2	2	2	1	4	1
3	1	2	3	2	2	4	1
0	0	3	2	1	2	3	2
3	3	0	3	1	2	3	1
3	1	1	4	1	2	3	1
0	1	2	1	3	1	2	1
1	1	1	2	2	2	3	1
1	1	2	1	2	2	4	1
2	3	1	4	3	4	3	1
4	1	2	2	2	3	4	1
1	2	2	2	1	1	4	1
0	0	1	1	2	2	4	1
2	2	2	2	2	2	3	1
3	1	2	3	3	3	1	1

2	3	2	2	4	3	1	1
3	2	1	4	3	4	2	1
4	2	2	2	4	3	1	2
1	1	1	4	1	1	4	1
2	1	2	3	1	1	4	1
3	2	2	2	4	4	2	2
0	0	1	2	2	2	3	1
2	2	1	1	2	2	3	1
0	0	0	1	1	1	1	1
1	1	1	3	3	3	2	1
1	1	2	1	1	4	4	1
0	1	0	3	2	1	2	1
1	1	2	3	1	1	4	1
2	2	2	4	1	2	4	1
2	3	1	2	1	2	4	1
1	1	2	1	2	2	4	1
3	3	3	2	2	3	3	2
0	0	1	3	2	2	3	1
3	2	2	4	2	2	4	1
1	3	1	2	1	2	1	1
3	1	2	1	2	2	4	1
3	2	2	4	4	3	3	1
0	0	1	1	1	1	3	1
3	1	1	3	1	1	4	1
3	1	1	2	2	3	2	4
3	2	2	2	1	1	4	1
4	1	1	3	2	3	3	4
3	2	0	3	2	2	3	1
1	1	2	2	1	2	3	1
1	1	1	1	2	2	4	1
2	1	2	3	1	3	3	1
2	1	1	1	2	3	3	1
2	1	2	2	2	2	3	1
3	1	2	3	2	2	3	1
2	1	2	3	2	2	3	1
2	1	1	3	1	3	4	1
2	0	0	2	2	2	4	1
2	1	1	4	1	3	4	1
2	1	1	3	1	1	4	1
3	0	2	3	2	2	3	2

1	1	1	3	1	1	4	1
2	2	1	1	2	3	3	1
3	1	2	1	2	2	3	1
2	1	2	3	1	2	3	1
1	1	1	2	1	3	3	1
3	1	2	2	1	2	3	1
2	1	2	3	2	2	3	1
2	1	1	2	2	2	4	1
2	0	1	1	2	2	4	1
3	1	2	4	1	2	4	1
2	1	2	3	1	3	4	1
2	1	1	1	1	1	4	1
2	1	1	1	1	1	4	1
3	2	1	4	4	4	2	1
1	1	1	1	1	1	4	1
1	1	1	3	2	2	3	2
0	0	0	4	4	1	1	1
2	1	1	1	2	1	4	1
1	1	1	2	1	3	4	1
2	1	2	1	1	4	4	1
1	1	1	1	1	4	4	1
2	1	1	4	1	2	4	1
3	2	1	4	2	3	1	2
3	2	2	3	3	3	1	1
3	1	1	3	1	2	4	1
3	2	1	3	3	3	2	1
2	1	2	2	2	2	4	1
2	1	2	1	3	3	2	1
2	1	1	1	2	1	4	1
2	1	1	4	3	3	2	1
3	2	2	4	2	2	4	1
3	2	1	4	3	3	2	1
1	0	1	1	1	1	4	1
3	4	1	2	2	2	4	1
3	1	2	4	1	2	3	1
3	2	1	2	1	1	3	1
2	2	1	4	2	3	3	1
2	1	1	2	1	1	3	1
3	1	1	2	2	2	2	2
1	1	1	3	1	2	4	1

2	1	1	2	1	1	3	1
2	2	1	1	2	3	3	1
3	1	2	4	3	2	2	1
2	1	2	4	3	2	2	1
2	1	0	1	1	2	4	1
1	1	1	1	1	1	4	1
2	1	2	1	1	2	3	1
3	1	2	2	2	3	3	1
3	1	2	1	3	2	3	1
3	1	2	3	2	3	3	1
3	1	2	3	2	2	3	1
2	2	1	4	3	2	2	1
2	1	0	3	2	1	3	1
2	1	1	2	1	1	4	1
2	1	1	1	2	1	3	1
2	1	1	3	2	3	3	2
2	1	3	3	2	2	3	1
3	2	1	4	3	3	2	2
3	1	2	3	2	3	3	1
3	2	2	2	3	2	4	1
2	1	1	3	1	2	3	1
3	1	2	1	1	2	4	1
2	1	1	3	2	1	3	1
3	2	2	3	2	3	3	2
1	1	1	3	1	2	3	1
3	2	1	3	3	3	2	1
0	0	0	1	1	1	4	1
3	2	2	3	2	3	3	1
1	1	1	2	2	1	4	1
2	1	1	2	2	1	3	1
1	0	0	1	1	2	3	1
2	1	1	2	1	1	4	1
2	2	1	4	2	2	3	1
1	0	1	2	2	1	4	1
2	2	1	3	3	2	3	1
2	1	1	3	3	2	3	1
2	2	2	4	3	3	2	1
3	1	1	3	2	2	3	1
0	0	1	3	1	2	3	1
2	1	1	3	3	1	3	2

3	1	1	1	2	2	3	1
2	1	2	2	2	2	3	1
3	1	1	2	2	2	4	1
2	1	2	2	1	2	3	1
2	1	2	2	2	2	3	1
3	2	2	3	1	2	3	1
3	2	2	4	3	3	2	2
3	2	2	4	3	3	2	2
1	1	1	2	2	2	3	1
2	1	1	3	2	1	3	1
2	2	1	3	4	3	1	1
2	1	1	2	1	1	3	1
2	1	1	2	2	3	3	1
2	1	1	2	2	1	3	1
1	1	1	2	1	1	3	1
1	1	1	2	1	2	3	1
3	2	2	4	4	3	2	1
1	2	1	3	2	2	3	4
2	1	1	2	2	2	3	1
1	0	1	2	2	2	3	1
1	1	2	2	1	2	4	1
2	1	1	2	2	2	3	1
1	1	1	2	2	2	4	1
3	2	2	3	3	3	2	1
3	2	2	4	4	3	1	1
3	2	2	4	2	2	3	2
1	0	1	1	2	2	3	2
3	1	1	4	3	3	2	1
3	1	2	4	3	2	2	1
3	2	2	3	3	2	2	1
3	2	2	3	3	3	2	1
1	1	2	2	2	2	3	1
3	1	2	3	3	2	3	1
2	1	1	2	2	2	4	1
3	1	2	4	3	3	2	1
3	2	2	3	3	3	2	1
1	1	2	3	3	3	2	1
3	2	2	4	3	3	2	1
1	1	1	2	2	2	4	1
1	1	1	2	2	2	3	1

2	1	1	2	2	2	3	1
3	2	2	4	3	3	1	1
1	1	2	2	2	2	3	1
1	1	1	2	2	2	3	1
3	2	3	4	3	3	2	1
1	1	1	2	2	2	4	1
2	1	1	2	2	2	4	1
2	1	1	2	2	2	3	1
3	1	1	4	3	3	2	1
2	0	0	2	2	2	3	1
2	0	1	2	2	1	3	1
3	1	1	3	3	3	1	1
2	1	1	3	3	2	1	2
1	1	1	2	2	2	4	1
1	1	1	3	3	3	2	1
1	1	1	2	2	1	4	1
3	2	1	3	3	3	2	1

TSK-6	TSK-7	TSK-8	TSK-9	TSK-10	TSK-11	TSK-12	TSK-13	TSK-14
3	3	3	3	1	1	3	2	1
1	2	3	3	2	2	3	3	2
2	2	2	2	2	3	2	4	4
3	3	3	1	2	3	3	3	1
4	4	4	4	4	4	3	4	4
3	1	2	3	2	2	3	3	3
1	1	2	3	2	1	3	3	1
3	3	4	3	1	3	3	3	4
2	2	2	1	1	2	3	3	1
1	1	3	2	3	1	3	4	1
3	3	4	2	4	3	3	3	2
1	1	2	3	1	1	3	4	2
2	1	3	2	1	3	3	3	2
2	2	3	1	1	2	3	3	3
2	2	1	1	1	2	1	1	1
1	3	3	1	3	2	4	3	1
3	3	2	3	3	2	2	2	3
2	2	4	2	2	2	3	3	2
3	3	3	3	2	3	4	3	1
2	2	1	1	3	2	4	4	2
2	1	2	3	3	3	3	2	2
1	1	3	1	2	3	3	4	3
2	1	2	2	2	3	3	4	1
2	1	3	3	3	3	4	3	1
2	2	2	2	4	2	3	4	2
2	2	3	2	2	2	3	2	2
3	2	2	4	3	3	2	4	3
2	1	2	3	3	2	2	3	3
2	2	3	2	2	3	3	3	2
2	2	2	1	4	3	3	4	1
3	2	3	3	2	3	3	3	3
1	1	4	1	3	1	3	3	1
3	2	2	3	2	2	3	2	2
3	2	2	3	3	3	2	4	4
2	1	3	2	2	3	3	4	2
1	2	2	1	1	1	3	2	1

4	1	1	1	1	1	4	4	1
3	2	2	2	3	3	2	3	1
2	2	2	1	1	3	4	3	1
3	1	2	2	3	3	4	3	1
3	2	3	1	2	4	3	3	2
2	2	3	2	3	2	2	3	2
1	1	3	1	3	3	3	3	1
2	2	2	3	3	3	3	3	4
2	1	2	2	2	3	3	3	3
1	3	2	2	3	1	3	4	1
2	3	3	2	3	3	3	3	3
2	1	2	2	3	3	3	3	3
3	1	2	2	3	3	2	3	2
2	2	1	2	2	3	2	2	1
2	2	3	2	3	2	3	2	2
2	1	2	2	2	3	2	4	3
2	1	3	2	1	3	2	3	2
4	4	2	4	4	4	1	4	4
3	1	4	2	2	2	3	4	1
1	1	1	1	1	1	2	2	1
2	3	3	2	3	2	3	3	3
3	2	2	2	3	2	3	3	2
3	3	2	2	3	2	3	3	2
3	2	1	2	2	3	3	4	2
2	3	3	3	2	2	4	3	1
2	3	2	2	2	3	3	4	1
2	2	1	1	1	4	4	4	2
2	3	4	2	4	2	3	4	3
2	3	3	3	3	3	3	3	3
1	2	3	2	3	2	3	3	2
3	1	3	1	3	3	3	3	3
3	2	3	1	4	3	4	4	1
2	2	3	1	2	3	3	3	4
3	3	2	2	2	3	2	3	3
3	1	2	2	3	4	2	3	3
3	3	2	2	3	3	3	3	2
3	1	2	1	1	3	3	3	1
1	1	2	1	2	3	3	3	1
2	3	2	3	2	3	3	2	2
2	2	3	2	2	3	3	3	1

2	2	2	1	2	3	3	3	1
3	2	2	3	2	4	2	3	3
3	1	2	1	2	3	3	3	2
2	3	1	3	2	2	2	2	4
1	1	2	2	2	3	4	2	1
1	1	2	2	2	2	3	4	1
3	2	1	1	3	3	3	3	3
2	2	3	2	3	3	3	3	2
4	1	2	2	3	3	2	3	3
3	1	2	2	3	3	3	2	2
3	1	2	2	2	3	2	3	4
2	2	2	1	3	2	4	3	1
3	1	2	2	2	2	3	2	3
2	1	1	3	1	1	3	3	1
2	1	2	2	1	3	3	3	3
3	1	2	1	2	4	2	3	2
3	1	2	2	1	3	3	4	1
2	3	1	2	3	3	4	2	4
3	3	2	1	2	3	3	3	3
2	1	2	2	1	3	3	2	3
2	2	2	2	2	3	3	3	2
3	2	2	1	3	3	3	3	3
3	2	2	2	3	3	2	2	3
3	3	2	3	3	2	3	3	3
2	2	3	1	3	3	3	4	1
1	2	1	1	2	2	3	4	1
3	3	1	3	1	3	3	3	3
2	1	2	2	2	4	3	4	1
2	3	3	1	3	3	2	2	2
2	1	2	3	1	1	3	2	1
3	2	2	3	4	3	2	4	4
2	3	1	1	2	3	3	3	1
2	2	2	1	4	3	3	4	1
2	1	2	2	1	3	4	4	2
3	3	2	4	4	2	2	3	3
3	2	2	1	1	4	3	4	2
1	1	2	1	2	3	3	4	1
1	1	2	1	1	3	3	4	1
2	1	2	1	4	4	4	4	2
3	2	2	3	3	3	3	4	2

2	2	2	1	4	4	3	2	4
4	3	1	3	3	3	4	2	4
3	3	1	1	4	4	3	3	3
1	2	2	2	2	2	3	3	2
1	3	1	1	1	3	3	4	2
4	3	2	3	4	4	3	3	3
1	2	2	3	3	3	3	3	2
4	3	1	2	4	4	2	4	4
1	1	1	1	1	1	1	1	1
3	2	3	3	3	2	2	2	3
3	3	2	1	1	3	3	4	2
1	2	1	3	2	1	3	3	2
2	1	2	2	3	3	4	4	1
2	2	3	1	3	3	2	3	2
3	1	1	2	3	3	3	3	1
3	3	1	1	3	3	3	3	1
3	2	2	2	2	3	3	3	3
2	2	2	2	1	3	3	2	2
3	2	2	2	3	3	3	3	2
2	2	2	2	1	2	1	2	2
2	1	1	2	4	3	4	4	1
2	1	2	2	4	4	3	3	2
1	1	2	2	1	4	3	3	1
2	1	1	2	3	3	4	4	1
3	1	3	2	3	2	2	2	2
2	1	1	4	4	3	3	3	4
2	3	3	3	3	4	3	4	2
3	3	3	2	4	4	3	4	2
1	2	1	2	2	3	3	3	1
2	1	2	2	2	3	3	3	2
3	3	1	3	4	3	4	3	2
2	1	2	1	2	3	3	3	1
1	2	2	2	3	3	3	3	2
2	2	2	1	3	3	3	3	3
2	1	2	1	3	3	4	3	2
2	1	1	2	3	3	4	2	1
2	3	2	3	2	1	1	2	1
1	3	1	3	3	3	4	3	1
2	3	1	2	3	3	4	3	3
3	2	2	3	4	4	4	4	1

1	1	1	2	3	3	4	4	1
2	2	1	2	2	3	4	3	2
2	2	2	1	3	3	3	3	2
3	1	1	2	4	3	4	4	2
2	1	1	1	3	3	3	4	1
2	2	2	2	3	3	3	2	3
1	1	1	3	2	1	4	4	1
1	1	1	2	3	3	3	4	2
1	1	2	1	3	3	3	3	1
1	1	3	1	3	3	4	3	3
1	3	1	2	2	3	4	3	1
1	1	2	1	2	3	4	3	1
1	1	2	1	3	3	4	3	1
3	3	1	3	4	4	2	3	3
2	1	1	1	3	3	4	3	1
3	3	2	2	4	2	3	3	2
1	3	3	3	3	2	2	2	2
1	1	2	1	1	3	4	3	2
2	1	3	1	2	3	4	3	1
1	1	2	1	3	4	4	3	1
1	2	1	2	2	3	4	4	1
2	1	1	3	1	4	3	4	1
1	1	1	3	4	4	2	3	1
2	2	2	3	3	3	2	3	3
1	1	1	3	2	4	4	3	1
3	1	2	2	3	3	4	3	2
2	1	3	2	2	4	3	3	2
1	1	1	3	4	4	3	2	2
1	1	2	1	2	4	4	3	1
2	2	1	3	4	3	3	3	3
3	1	1	3	3	3	3	3	2
3	1	1	3	3	3	4	4	2
2	2	2	1	2	3	4	4	1
1	2	1	3	4	4	3	3	2
2	1	2	3	3	4	4	3	3
1	2	2	2	3	3	4	3	2
2	2	2	3	4	3	3	3	2
2	2	1	2	3	3	3	1	2
2	2	3	2	2	2	3	2	2
2	2	1	3	2	3	3	3	2

2	1	2	1	4	3	4	3	2
2	1	1	1	3	4	3	3	2
1	2	2	3	4	3	3	3	2
1	1	1	3	2	4	4	2	1
2	1	2	2	4	4	3	2	1
2	1	2	2	1	4	4	2	1
1	1	4	2	3	3	4	2	2
1	1	2	1	4	3	3	2	2
1	1	2	1	3	3	3	3	2
1	1	1	3	3	3	3	2	2
1	2	2	3	4	4	4	2	3
1	1	1	2	3	3	4	3	2
2	2	1	2	3	3	3	3	2
1	1	2	1	3	3	4	4	1
2	1	1	1	3	3	4	3	2
3	3	2	3	3	2	3	2	3
3	2	1	3	4	4	4	2	3
2	2	2	3	4	3	3	3	2
2	2	2	3	3	3	3	2	2
2	1	3	3	4	3	3	3	3
2	2	1	1	3	4	3	3	2
2	2	2	1	3	3	4	3	2
1	1	1	3	3	3	3	3	3
2	1	2	3	4	4	3	3	3
1	1	2	2	3	3	2	3	2
3	1	2	3	4	4	3	3	3
1	2	4	1	4	2	3	4	2
2	2	2	3	4	4	3	3	3
2	2	2	2	3	3	4	3	2
1	1	1	2	3	3	4	3	2
1	1	2	1	2	3	4	4	1
1	1	2	1	3	3	4	3	2
2	1	2	4	3	3	3	3	3
2	2	2	1	2	3	4	3	2
2	2	2	3	4	3	3	4	3
2	1	2	3	3	3	3	3	2
2	2	2	3	3	3	3	3	3
1	3	1	2	3	3	4	3	2
2	2	3	2	3	2	3	2	2
3	3	2	3	4	1	3	3	2

2	1	2	1	2	3	4	3	2
2	2	2	2	3	3	3	3	2
2	2	2	2	3	3	3	3	2
1	2	3	2	3	3	3	3	2
1	2	2	3	3	3	3	2	2
2	2	2	3	3	3	4	3	2
3	2	1	3	3	3	3	2	3
2	3	2	3	3	3	3	2	3
2	1	2	1	2	3	3	3	2
1	1	2	2	2	3	4	3	2
2	3	2	3	4	3	3	3	2
2	2	2	2	3	3	4	3	1
1	1	2	1	4	3	4	3	2
2	1	2	2	3	2	3	3	2
2	1	1	1	2	3	4	4	2
1	1	3	1	3	3	3	3	2
3	3	2	4	4	4	3	4	3
2	3	3	3	2	1	1	3	2
2	2	1	2	2	3	3	3	2
2	1	2	2	2	3	3	3	2
2	1	1	2	2	3	4	3	2
2	2	2	2	3	3	4	3	2
1	1	2	2	2	3	3	3	2
3	2	2	3	4	4	3	4	3
3	3	1	3	3	3	3	3	4
2	2	1	2	3	3	3	3	2
2	3	4	2	4	2	3	4	3
2	2	1	3	3	3	3	3	3
2	1	1	3	4	4	2	3	3
3	2	2	3	3	4	3	3	3
3	2	1	2	3	4	3	3	3
2	1	2	2	3	3	3	3	1
2	1	2	3	3	3	3	3	3
2	1	2	2	3	3	4	3	1
2	2	2	3	4	3	2	3	3
2	2	2	3	3	3	3	3	3
2	2	2	3	4	4	3	3	3
3	1	1	3	3	4	3	3	3
2	1	2	1	2	3	4	3	2
2	1	2	2	3	3	4	4	2

2	1	3	1	2	3	4	4	1
3	2	2	3	3	4	2	3	3
2	1	2	1	3	3	3	3	2
2	1	3	2	3	3	4	3	1
3	1	1	3	4	4	2	3	3
2	1	1	1	3	3	4	3	2
1	1	1	1	2	3	4	3	1
1	1	2	2	3	3	4	3	1
3	2	2	3	4	4	3	3	3
3	1	1	2	2	2	3	3	2
2	1	3	2	3	2	3	3	2
3	2	2	3	4	4	2	3	3
3	3	3	3	2	1	1	1	1
2	1	2	2	3	3	3	3	2
3	1	3	2	4	4	2	3	3
2	1	2	1	3	3	4	3	1
2	2	1	3	4	4	2	3	3

TSK-15	TSK-16	TSK-17	ESES-1	ESES-2	ESES-3	ESES-4	ESES-5	ESES-6
3	4	1	3	4	4	3	3	1
2	3	2	2	2	4	2	4	2
3	3	1	3	2	2	2	2	1
2	3	2	3	3	3	3	2	1
3	4	4	4	4	3	4	4	4
1	2	3	3	4	3	2	4	3
1	2	1	3	3	3	3	3	3
1	3	3	2	4	2	3	2	1
1	2	2	3	3	3	2	3	1
1	1	2	3	3	2	2	2	2
3	2	1	3	3	2	3	3	1
2	3	2	3	3	3	3	2	2
2	3	2	4	4	3	3	3	2
2	1	1	4	3	3	2	3	2
2	1	1	1	1	1	1	1	1
2	3	2	3	3	3	3	3	3
3	2	3	2	3	3	3	2	2
3	2	2	3	3	3	2	2	2
2	3	3	3	2	4	3	4	3
1	2	3	3	3	2	2	2	2
1	2	1	4	4	3	3	2	2
3	2	2	3	2	3	3	2	2
2	3	1	4	4	4	3	3	2
2	3	1	4	3	3	3	2	2
1	3	1	3	4	4	3	2	2
2	2	1	3	3	3	2	1	1
4	2	3	3	2	2	3	1	1
3	2	1	2	2	3	2	1	1
2	3	1	3	3	2	2	1	1
2	2	2	4	4	4	3	3	4
3	2	1	3	2	3	2	1	1
3	2	1	3	3	3	1	1	2
3	3	2	3	3	3	3	2	2
4	3	1	2	2	1	2	1	1
4	3	2	3	3	2	2	1	1
2	2	1	2	3	3	3	3	3

1	4	1	4	4	4	4	1	4
3	3	2	3	2	2	3	1	1
2	4	1	3	3	3	3	1	1
2	2	1	3	3	2	2	1	1
2	2	1	3	2	3	3	1	1
2	3	2	3	3	1	3	2	2
1	1	1	2	4	4	3	3	3
3	1	3	2	2	3	3	1	1
2	3	2	3	2	2	3	1	1
1	3	3	3	4	4	3	3	3
3	1	2	2	3	3	2	2	3
3	2	1	3	3	2	2	1	1
1	2	1	3	2	2	3	1	1
2	2	2	3	2	2	3	1	1
2	2	2	3	3	3	4	2	1
2	1	2	2	3	2	3	1	1
2	3	2	3	3	3	3	1	1
4	4	4	4	4	1	2	4	4
2	2	1	3	3	2	3	1	1
1	1	1	2	2	3	3	3	3
2	2	3	2	3	3	3	2	2
3	3	2	3	3	3	2	3	3
2	2	2	3	3	3	3	3	3
2	3	2	2	2	2	2	1	1
3	3	2	3	3	3	3	3	3
2	2	1	3	3	2	3	1	1
4	1	1	2	4	4	4	1	1
3	4	1	4	4	4	4	3	3
3	1	3	3	3	3	3	3	3
2	3	1	3	2	3	2	1	1
1	2	1	3	3	3	3	1	1
3	3	1	3	3	3	2	1	1
3	2	1	3	3	2	3	1	1
3	3	3	2	2	2	2	2	2
2	2	1	2	3	3	3	1	1
4	3	2	3	2	2	3	1	2
2	3	2	2	3	3	3	1	1
2	3	1	3	3	3	3	1	1
2	3	2	3	3	4	3	3	4
2	3	1	2	3	2	2	1	1

2	2	2	3	2	2	3	1	1
2	3	1	3	2	2	2	1	1
3	2	1	3	2	2	3	1	1
3	2	2	2	2	2	2	1	1
1	2	1	3	3	3	3	2	2
3	3	1	3	3	3	3	1	1
2	3	2	2	2	2	2	1	1
1	2	1	3	3	3	2	2	2
1	2	1	2	2	2	2	1	1
1	2	2	2	2	2	3	1	1
4	1	2	2	2	2	2	1	1
3	3	2	3	3	3	3	3	2
3	2	2	2	2	2	2	1	1
3	3	3	2	3	2	4	1	2
3	3	1	3	2	2	3	1	1
2	3	1	3	2	2	3	1	1
2	2	1	3	2	2	2	1	1
2	3	3	3	3	2	3	1	2
1	2	1	3	2	1	3	2	2
2	1	1	3	2	2	3	1	1
2	3	1	3	2	2	3	1	1
2	2	1	2	2	2	2	1	1
2	2	2	2	2	2	2	1	1
3	2	3	3	3	3	3	3	3
2	3	1	3	3	3	2	1	1
1	1	1	3	3	2	3	2	2
2	2	1	3	3	3	3	1	1
2	1	1	3	3	3	3	1	1
3	3	2	3	3	3	3	3	3
1	1	1	3	3	2	4	3	3
3	1	1	3	2	2	2	1	1
2	3	1	3	2	2	3	1	1
3	3	2	2	1	1	2	1	1
2	2	1	3	2	2	3	1	1
3	2	4	3	3	2	2	2	2
2	2	1	3	2	2	3	1	1
1	3	1	3	2	2	3	1	1
2	3	1	3	2	2	3	2	2
2	2	1	3	2	2	3	1	1
3	1	2	3	1	1	3	1	1

3	2	2	3	1	1	3	1	1
4	1	2	2	1	1	2	1	1
3	1	2	3	2	2	3	1	1
3	2	1	3	4	4	3	2	2
2	2	2	3	2	2	3	1	1
2	2	1	3	3	3	3	2	2
3	2	2	3	4	4	3	3	4
4	1	2	3	1	1	3	1	1
1	4	4	3	3	3	3	3	3
3	2	3	2	2	2	3	3	3
2	3	1	3	2	2	3	1	1
2	1	2	3	3	2	2	1	1
1	2	1	3	2	2	3	1	1
2	1	1	3	2	2	3	2	2
2	3	1	2	1	1	3	1	1
2	2	1	2	1	1	2	1	1
3	3	2	3	3	3	3	3	3
1	1	2	4	3	3	4	3	3
3	1	2	2	1	1	2	1	1
1	2	2	3	3	3	2	3	3
2	4	1	3	3	3	3	3	3
2	2	2	3	2	2	3	1	1
1	1	1	3	3	3	3	1	1
1	2	1	3	3	3	3	2	2
3	3	2	2	2	2	2	2	2
3	1	2	3	3	3	3	1	1
2	3	2	3	4	3	3	2	3
4	4	1	3	3	3	2	2	2
2	2	1	3	3	2	3	2	2
2	2	2	3	1	1	3	2	2
2	2	1	3	2	2	3	2	2
2	2	1	3	2	2	3	2	2
2	2	1	3	2	2	3	1	1
2	2	2	3	2	2	3	2	2
2	2	1	3	2	2	3	2	2
2	2	1	3	2	2	2	1	1
1	1	1	4	4	4	4	1	1
2	1	1	3	3	3	3	2	2
3	2	1	3	3	3	3	2	2
1	4	1	3	4	4	3	4	4

2	2	1	3	2	2	3	2	2
3	1	2	3	3	3	3	2	2
2	1	1	3	3	3	3	2	2
2	2	1	3	3	3	3	2	2
2	2	1	2	3	3	3	1	1
2	2	3	3	2	2	2	1	1
1	3	1	3	3	2	3	2	3
2	1	1	3	2	2	3	2	2
1	2	1	3	2	2	3	3	3
1	2	1	3	3	3	3	3	3
2	1	1	3	2	2	3	2	2
1	2	1	3	1	3	3	1	1
1	2	1	3	1	1	3	2	2
3	2	1	1	2	2	2	1	1
1	1	1	3	3	3	3	2	2
3	1	2	3	3	3	3	2	3
3	2	3	3	3	2	2	2	3
2	2	1	3	3	3	3	2	2
1	2	1	3	2	2	3	2	2
1	2	2	3	3	3	3	2	2
1	1	1	3	3	3	3	2	2
1	1	1	3	3	3	2	1	1
2	1	1	3	2	1	2	1	1
2	2	2	3	2	3	3	2	2
2	3	1	3	3	3	3	1	1
1	2	1	3	2	2	3	1	1
2	2	1	3	2	2	3	1	1
2	2	2	2	1	1	2	1	1
2	2	1	3	3	3	3	1	1
3	1	2	3	2	2	2	1	1
2	1	1	3	3	3	2	1	1
2	2	1	3	1	1	2	1	1
1	2	1	3	3	3	3	2	2
2	2	1	3	2	2	3	1	1
2	1	1	2	2	2	1	1	1
2	2	1	3	3	3	3	2	2
3	1	1	3	2	2	3	1	1
2	1	2	3	3	3	3	2	2
2	2	2	3	3	3	3	3	3
2	2	1	3	3	3	3	2	2

2	2	1	3	3	3	3	2	2
2	1	2	3	3	3	3	2	2
3	2	2	3	2	2	3	1	1
3	1	2	3	2	2	3	1	1
3	2	2	3	3	3	2	2	2
2	2	2	3	2	2	3	2	2
2	1	1	3	3	3	3	2	2
2	2	2	3	3	3	3	2	2
2	2	2	3	3	3	3	2	2
2	2	2	3	3	3	3	2	2
2	2	2	4	2	2	3	1	1
2	2	1	3	2	2	3	2	2
2	2	1	3	3	3	3	2	2
1	2	1	3	3	3	3	2	2
2	2	2	3	3	3	3	2	2
3	3	3	3	3	2	2	2	2
2	2	2	3	2	2	3	1	1
3	2	2	3	2	2	3	1	1
2	2	2	3	2	2	3	1	1
3	1	2	2	2	2	2	1	1
3	3	2	3	2	2	3	1	1
2	2	2	3	2	2	3	2	2
2	2	2	3	2	2	3	1	1
2	1	2	3	2	2	3	1	1
2	2	2	3	3	3	3	2	2
3	1	2	3	2	2	2	1	1
2	3	2	4	4	4	4	2	2
3	2	2	2	2	2	2	2	2
2	2	2	3	2	2	2	1	1
2	2	1	3	3	3	3	3	3
1	1	1	3	3	3	3	2	2
2	2	1	3	3	3	3	3	3
2	2	2	2	3	3	2	1	1
2	2	1	3	3	3	3	2	2
3	2	2	2	2	2	2	2	2
3	2	1	2	2	2	2	1	1
3	2	2	3	2	2	2	1	1
2	2	1	3	2	2	2	1	1
2	3	2	3	3	3	3	2	2
3	2	2	3	3	2	3	2	3

3	2	1	3	2	2	3	2	2
2	1	1	3	2	2	3	3	3
2	2	1	3	3	3	3	2	2
2	2	1	3	3	3	3	3	3
2	2	1	3	3	3	3	2	2
2	3	2	2	2	2	2	1	1
3	2	2	2	2	2	2	1	1
3	1	2	2	2	2	2	1	1
2	2	1	3	3	3	3	2	2
1	3	1	3	3	3	3	2	2
3	1	1	3	3	3	2	1	1
2	2	1	3	3	3	3	2	2
2	2	2	3	3	3	3	2	2
2	2	1	3	2	2	3	2	2
2	2	1	3	3	3	3	3	3
2	3	1	3	2	2	3	2	2
3	2	2	2	2	2	2	1	1
3	3	1	3	3	4	3	2	3
2	2	1	3	2	2	3	2	2
2	2	1	3	3	3	3	2	2
1	2	1	3	3	3	3	3	3
2	3	1	3	3	3	3	2	2
2	2	1	3	3	3	3	2	3
3	2	2	3	2	2	3	1	1
4	2	2	3	2	2	3	1	1
3	1	1	2	2	2	2	1	1
3	4	1	4	4	4	4	3	3
3	2	2	2	2	2	2	1	1
3	3	2	3	2	2	3	1	1
3	2	2	3	2	2	3	1	2
3	2	2	3	2	2	2	2	2
1	2	1	4	3	3	4	2	2
3	2	1	3	2	2	3	1	2
1	2	1	3	2	2	3	1	2
3	2	2	3	2	2	3	2	2
3	2	2	2	2	2	2	1	1
3	2	2	3	2	2	2	1	1
3	2	2	2	2	2	2	1	1
2	2	1	3	3	3	3	2	2
2	3	1	3	3	3	3	2	2

1	2	1	4	3	3	3	2	2
2	2	2	2	2	2	2	1	1
2	2	1	3	2	2	3	2	2
1	3	1	4	3	3	3	2	2
3	2	2	2	2	2	2	1	1
2	3	1	4	3	3	3	2	2
1	2	1	4	3	3	3	2	2
1	3	1	4	3	3	3	2	2
3	2	2	3	2	2	2	2	2
1	1	1	3	2	2	3	2	2
2	1	1	3	2	2	3	2	2
3	2	3	3	2	1	3	2	2
2	2	2	3	2	2	3	2	2
2	3	1	4	3	3	3	2	2
2	2	2	3	2	1	3	1	1
2	1	1	3	2	2	3	2	2
3	2	2	2	2	2	2	2	2

ESES-7	ESES-8	ESES-9	ESES-10	APGAR-1	APGAR-2	APGAR-3	APGAR-4
1	3	3	3	2	2	1	2
2	1	3	3	2	2	2	2
1	2	2	2	2	1	2	2
2	2	2	2	2	2	2	1
4	4	4	4	1	1	1	1
2	3	3	4	1	1	1	1
3	3	3	3	2	2	2	2
2	3	4	3	2	1	1	2
3	3	3	3	1	2	2	1
2	2	3	3	1	1	2	1
2	2	3	3	2	2	1	1
2	3	4	3	2	1	1	2
3	3	3	3	1	2	2	1
3	3	3	3	2	2	2	1
1	1	1	1	2	2	2	2
3	3	3	3	2	2	2	2
2	2	2	2	2	2	2	2
3	3	3	3	2	1	2	2
3	4	3	1	1	2	1	2
2	1	1	1	1	1	2	1
3	3	2	2	2	2	2	0
2	2	2	2	2	2	2	1
3	2	3	2	2	2	1	2
1	2	2	2	2	2	1	1
2	2	3	2	2	1	1	2
2	2	2	3	2	1	1	2
2	3	2	2	2	1	2	1
1	2	2	2	2	2	1	1
1	2	2	2	2	2	1	1
4	4	4	4	2	2	2	2
2	2	2	2	2	2	1	1
1	2	3	1	2	2	2	2
3	3	3	3	1	1	1	1
1	1	2	2	1	1	1	1
1	2	2	2	2	1	2	1
2	1	1	3	2	2	2	2

3	4	4	4	2	2	2	2
2	1	2	2	2	1	1	1
1	2	2	2	2	0	1	2
2	1	1	2	2	1	2	1
2	2	2	2	2	1	2	1
1	2	2	2	1	2	1	1
3	3	3	3	1	1	1	1
2	2	2	2	2	0	1	2
2	3	1	1	0	1	2	1
3	3	3	2	2	2	2	2
3	3	3	3	1	1	2	1
2	2	2	2	1	1	1	1
2	2	2	2	1	0	1	1
2	2	2	1	2	0	1	0
3	3	3	3	2	2	1	1
2	2	2	2	2	1	2	1
2	2	2	2	2	1	2	2
4	4	1	3	2	1	2	1
2	2	2	2	2	1	2	1
3	3	3	3	2	2	2	2
2	2	2	2	2	2	2	2
3	3	3	3	2	1	2	2
3	3	3	3	1	1	1	2
2	2	2	2	1	1	1	1
2	2	3	3	2	2	2	2
2	2	2	2	1	2	2	1
4	3	4	3	2	2	1	2
3	2	3	2	2	2	2	2
2	2	3	3	2	2	1	0
2	2	2	1	2	1	2	1
1	2	2	1	1	1	2	2
2	2	2	2	2	1	2	2
2	3	1	1	2	1	2	2
2	2	2	2	2	1	1	2
1	2	2	2	1	1	2	1
2	2	2	3	2	1	2	2
3	3	2	2	2	1	2	1
3	3	2	2	2	2	2	1
3	3	3	3	2	2	2	2
2	2	2	2	2	2	2	1

2	2	2	2	2	2	2	1
1	1	1	2	2	1	2	1
1	2	2	2	1	2	1	1
1	2	1	1	2	2	2	0
3	3	3	3	2	2	2	1
2	2	2	2	2	1	2	1
1	1	2	1	2	1	2	1
3	3	3	3	1	1	1	1
1	2	1	1	2	1	1	1
2	1	2	1	1	1	1	1
2	2	2	3	2	1	1	1
3	3	4	4	1	1	2	1
1	2	2	2	1	1	2	2
2	3	3	3	1	2	1	1
2	2	2	3	1	1	2	1
2	2	2	1	2	1	2	1
2	2	1	2	1	1	2	1
3	3	2	2	1	1	1	1
2	2	2	3	1	1	2	1
2	3	2	3	1	1	1	2
2	2	3	3	2	2	2	1
2	2	1	2	2	1	2	1
1	2	1	2	2	1	2	1
3	3	3	3	2	2	2	2
2	2	2	3	2	1	1	2
2	2	2	3	2	2	2	1
2	2	2	2	2	1	2	1
2	2	2	3	2	1	2	1
3	3	3	4	2	2	2	2
3	3	4	4	1	2	2	2
2	3	2	3	2	1	2	1
2	2	2	3	2	2	2	1
2	2	2	2	1	1	1	1
2	2	2	3	2	1	2	1
2	2	3	2	2	2	2	2
2	2	2	3	1	1	2	1
2	3	3	1	2	1	2	1
3	3	2	3	1	1	1	1
2	3	2	3	2	1	1	1
2	3	2	2	1	1	1	1

2	2	2	1	1	1	1	1
2	2	2	1	1	1	1	1
2	2	2	3	1	1	1	1
3	3	3	3	2	2	1	1
2	2	2	3	2	1	1	2
2	3	3	3	0	1	1	0
3	3	3	3	2	2	2	2
2	2	2	2	1	1	1	1
3	3	3	3	2	2	2	2
2	3	3	3	1	1	1	1
2	2	2	2	1	1	1	1
1	2	1	2	2	2	2	2
2	2	2	3	1	1	1	1
2	2	2	3	1	1	1	1
2	2	2	2	2	1	1	1
2	2	2	3	1	1	1	1
3	3	3	3	2	2	2	2
3	4	3	4	2	1	1	2
2	2	2	3	2	1	2	1
3	3	3	3	0	0	2	0
3	3	2	2	2	2	2	1
1	1	1	2	2	2	2	1
3	3	3	2	2	2	2	1
3	3	2	3	2	1	2	1
2	2	2	2	1	1	2	2
2	2	2	3	2	1	1	1
3	3	3	3	2	2	2	2
2	3	3	2	2	2	2	1
2	2	2	3	2	1	2	1
2	2	2	2	1	2	2	2
2	2	2	3	1	1	1	1
2	2	2	3	2	1	2	1
2	2	2	3	2	1	2	2
2	3	1	3	2	1	2	1
1	2	2	3	2	1	2	1
2	2	2	3	2	1	2	1
4	4	4	4	2	2	2	2
3	3	3	3	2	1	2	1
2	2	2	3	2	2	2	1
4	4	4	4	2	2	2	2

2	2	2	3	2	2	2	1
3	3	3	3	2	1	2	1
3	3	3	2	2	1	2	1
2	3	3	3	2	1	2	1
2	2	2	3	2	2	2	1
1	2	2	3	2	1	2	1
3	3	3	3	2	2	2	2
2	2	2	3	2	2	2	1
2	3	3	3	2	1	2	1
2	2	2	3	2	2	2	1
2	3	3	3	2	2	1	1
1	3	3	3	1	1	2	1
2	2	2	3	2	1	2	1
1	1	1	1	2	1	1	1
2	2	1	3	2	1	2	1
3	3	3	3	2	1	2	2
3	3	3	3	2	2	2	2
3	3	3	3	2	1	2	2
3	3	3	3	2	2	1	1
3	3	2	3	2	2	2	1
2	2	3	3	2	2	2	2
1	2	2	3	2	1	2	1
2	3	1	3	0	1	1	0
2	3	2	3	2	1	2	1
2	3	3	3	2	2	2	1
2	2	2	3	2	1	1	1
2	1	1	2	2	1	1	1
1	1	2	2	1	1	1	1
2	3	2	3	2	2	1	1
2	2	2	3	1	1	1	0
2	2	3	3	1	1	2	1
2	2	2	2	1	1	1	1
3	3	3	4	2	2	1	1
2	2	2	4	2	1	1	1
2	2	3	3	2	1	1	1
3	3	2	4	2	1	1	1
2	2	2	3	1	1	1	1
2	2	3	3	2	1	1	1
3	3	3	3	2	2	2	2
3	3	3	3	2	1	2	1

3	3	3	3	2	1	1	1
3	3	3	3	1	1	1	1
2	2	3	3	2	1	1	1
3	3	3	3	2	1	1	1
2	2	2	3	2	1	1	1
3	3	3	3	2	1	1	1
3	3	3	3	2	1	1	1
2	3	3	3	2	1	1	1
2	2	2	2	2	1	1	1
3	3	3	3	2	1	1	1
3	2	2	4	2	1	1	2
2	2	2	3	1	1	1	1
3	3	3	4	1	1	1	1
3	3	3	4	2	1	1	1
3	3	3	4	1	1	1	1
2	2	2	2	1	1	2	1
2	2	2	2	2	1	1	1
2	2	2	3	1	1	1	1
2	2	2	3	1	1	1	1
2	2	2	3	1	1	1	1
2	3	3	3	2	1	1	1
3	3	3	3	1	1	1	1
2	3	3	3	1	1	1	1
2	2	2	3	2	1	1	2
3	3	3	3	1	1	1	1
2	2	2	3	2	1	1	1
4	3	4	4	2	2	2	2
2	2	2	3	2	1	1	1
2	2	2	3	1	1	1	1
3	3	3	4	2	1	1	1
3	3	3	4	1	1	1	1
3	3	3	4	1	1	1	1
2	2	2	3	2	1	1	1
3	3	3	3	2	1	1	1
2	2	2	3	2	1	1	1
2	3	3	2	1	1	1	1
3	2	2	3	1	1	1	1
2	2	3	3	2	1	1	1
3	3	3	3	2	2	2	2
2	3	3	3	1	2	2	1

2	3	3	3	1	1	1	1
3	3	3	3	2	2	1	2
3	3	3	4	2	1	1	1
3	3	3	4	2	1	2	1
3	3	3	4	2	1	2	2
3	3	2	3	1	1	1	1
3	3	2	3	1	1	1	1
1	2	3	3	1	1	1	1
3	3	3	3	1	1	1	1
3	3	3	3	2	1	2	2
2	3	2	2	1	1	1	1
3	3	3	3	0	1	1	0
3	3	3	3	2	1	1	1
3	3	3	4	1	1	1	1
3	3	3	4	2	2	2	1
3	3	3	2	1	1	1	1
2	2	2	3	1	1	1	1
3	2	3	3	2	2	2	2
3	3	3	4	1	1	1	1
2	3	3	4	2	1	1	1
3	3	3	4	2	1	1	1
3	3	3	4	1	1	1	1
3	3	3	4	1	1	1	1
2	3	3	3	1	1	1	1
2	2	3	4	1	1	1	1
3	3	2	3	2	2	1	2
3	2	3	2	2	2	2	2
2	2	2	3	1	1	1	1
2	2	2	3	2	1	1	1
2	2	2	3	2	1	1	1
2	2	2	3	1	1	1	1
2	3	3	4	2	1	1	1
2	3	2	3	1	1	1	1
2	3	3	4	2	2	2	1
2	2	2	4	1	1	1	1
2	2	2	3	2	2	1	1
2	3	2	4	2	1	1	2
2	2	2	4	1	1	1	1
3	3	3	4	2	2	2	2
2	3	3	4	2	1	2	2

2	3	3	4	2	2	2	1
2	2	3	4	1	2	1	1
2	3	3	4	2	1	2	1
2	3	3	4	2	1	1	2
1	3	3	3	2	2	2	1
2	3	3	4	1	2	2	1
2	3	3	4	2	2	2	1
3	2	2	4	2	2	2	2
2	2	2	3	1	1	1	1
2	2	2	3	1	1	1	1
3	3	2	3	1	1	1	1
2	2	2	3	1	1	1	1
2	2	2	3	2	2	2	2
2	3	3	4	2	2	2	2
2	2	2	3	1	1	1	1
3	3	3	3	1	1	1	1
2	2	2	3	1	1	1	1

APGAR-5	PCS-1	PCS-2	PCS-3	PCS-4	PCS-5	PCS-6	PCS-7	PCS-8
2	1	2	1	2	1	3	4	4
2	1	2	0	0	0	0	0	1
1	3	0	3	2	3	3	3	3
2	1	0	1	1	2	1	2	1
1	2	2	2	3	3	3	3	3
1	1	2	3	0	0	1	0	2
2	1	0	1	0	1	1	0	1
2	2	3	0	1	0	1	0	3
2	1	0	0	0	1	2	2	3
1	1	1	0	1	1	1	0	4
2	2	1	1	2	4	1	1	3
2	1	0	0	1	1	1	0	0
2	3	2	1	1	0	1	2	3
2	1	1	0	0	1	1	1	2
2	0	0	0	0	0	0	0	0
2	1	0	0	0	1	1	1	3
1	2	1	1	1	1	1	1	4
2	1	0	0	1	0	1	0	1
2	2	3	0	4	1	3	0	4
2	3	2	2	2	1	3	2	4
2	0	1	0	1	0	1	1	1
2	0	1	0	1	0	2	0	3
2	1	0	1	0	1	2	1	2
2	1	2	1	2	2	1	1	3
2	3	1	1	1	1	2	1	3
2	0	1	1	1	1	2	1	4
2	3	2	1	2	1	2	1	3
2	3	3	1	2	3	4	2	4
2	2	2	1	1	2	4	2	4
2	0	0	0	0	1	0	0	4
2	3	3	1	1	2	4	2	4
2	2	0	0	0	1	0	0	1
1	2	2	1	1	1	1	2	1
1	4	3	2	2	4	4	2	4
7	3	3	2	2	3	4	2	4
2	1	1	0	0	0	2	1	1

2	0	0	0	0	0	0	0	2
1	4	3	4	3	4	4	4	4
2	3	2	2	1	2	4	2	4
2	3	2	1	1	3	4	2	4
2	2	3	3	2	2	4	2	4
2	0	1	2	2	1	2	1	3
1	1	1	0	0	0	0	1	0
2	2	2	2	1	3	4	3	4
2	3	4	2	3	4	3	2	4
2	1	1	2	2	1	1	0	2
1	2	1	3	1	1	2	4	4
2	4	3	2	2	3	3	2	4
2	2	4	2	2	3	2	2	4
2	3	2	1	1	3	4	2	4
2	0	0	1	0	3	0	1	3
2	2	1	0	0	1	3	1	3
2	1	1	0	0	1	3	0	4
1	1	4	4	0	4	4	4	4
2	2	2	1	1	4	3	2	4
2	0	0	0	0	0	1	0	0
2	0	0	0	1	1	2	3	1
2	2	1	1	1	2	4	1	4
2	1	1	0	0	0	0	1	1
2	1	2	1	1	2	2	1	4
2	1	1	2	1	3	3	3	3
2	3	2	3	2	4	3	2	4
2	4	4	1	2	3	4	2	4
2	4	3	2	2	4	3	2	4
1	3	3	3	3	3	3	3	3
2	4	3	2	3	4	4	4	4
2	4	3	4	2	3	4	3	4
2	3	4	3	3	4	4	3	4
2	4	4	3	2	3	4	3	4
2	2	2	2	2	2	2	2	2
2	4	3	2	2	4	3	2	4
2	1	2	2	1	2	3	2	2
2	3	1	0	0	0	3	1	4
2	2	1	1	1	1	2	1	3
2	0	0	0	0	0	0	0	0
2	1	0	0	1	1	1	0	1

2	3	4	2	3	4	4	3	4
2	4	3	2	2	4	4	3	4
2	4	4	3	1	3	3	3	4
2	4	4	2	3	4	4	3	4
2	4	1	2	2	3	4	3	4
2	0	1	0	0	2	1	2	2
2	4	3	2	2	4	4	4	2
2	0	1	0	0	0	1	0	2
2	4	3	2	2	3	4	3	4
2	4	3	2	2	3	4	4	4
2	4	4	2	2	4	4	2	4
1	1	2	1	1	2	3	0	4
2	4	4	2	2	3	4	3	4
2	3	1	1	3	1	3	1	3
2	3	3	3	3	3	4	2	4
2	3	3	2	2	4	3	2	4
2	4	2	2	2	4	4	3	4
2	3	2	2	1	2	2	2	3
2	2	2	1	1	2	2	0	2
2	4	3	2	3	3	4	2	4
2	1	1	0	0	1	2	1	2
2	4	3	3	2	3	4	3	4
2	4	3	3	2	3	3	3	4
2	3	1	1	1	1	2	2	2
2	4	3	2	2	3	4	4	4
2	2	2	0	0	0	1	0	0
2	4	3	2	3	2	4	3	3
2	4	3	1	2	2	4	4	4
2	1	1	2	1	0	1	2	0
2	2	3	2	4	2	0	0	4
2	4	3	4	3	3	4	3	4
2	4	2	0	2	3	3	2	3
1	4	3	2	3	3	4	4	4
2	3	1	2	1	1	3	1	4
2	3	2	2	3	2	3	3	3
2	4	3	3	1	2	4	2	4
2	3	1	0	0	1	1	1	2
1	3	0	0	1	2	3	3	3
2	4	2	1	2	3	4	3	4
1	4	3	3	2	3	3	3	3

1	1	1	1	1	2	3	1	1
1	4	4	3	3	4	4	4	4
1	4	3	3	2	2	4	3	3
1	1	0	0	0	0	0	0	4
2	1	2	0	1	2	2	1	2
1	4	3	3	3	3	3	4	3
2	0	1	1	0	0	1	1	1
1	4	4	4	3	4	4	4	4
2	1	0	0	0	0	0	0	0
1	1	2	2	1	2	3	3	3
1	2	1	1	0	2	2	1	2
2	1	1	1	1	1	1	1	1
1	3	3	1	0	2	2	0	2
1	2	1	2	1	2	2	0	2
2	4	4	3	2	4	4	3	4
1	0	1	0	0	1	0	0	0
2	2	2	2	2	2	2	2	2
2	1	2	2	0	0	1	2	1
2	2	3	2	2	3	4	3	4
2	1	0	1	0	0	1	3	3
2	4	3	4	3	4	4	3	4
2	4	4	4	3	4	3	4	4
2	0	1	0	0	1	0	0	0
2	3	1	2	1	1	2	2	3
2	3	3	3	3	3	3	3	3
2	3	4	4	3	4	4	3	4
2	2	1	2	1	1	1	2	3
2	3	2	2	3	3	3	3	3
2	0	0	0	0	0	0	1	0
2	2	1	1	2	1	2	1	2
2	4	3	3	3	2	3	3	3
2	4	4	4	2	4	4	4	3
2	4	3	2	2	4	4	4	4
2	2	2	1	2	2	3	2	4
2	4	4	3	2	3	3	3	4
2	4	4	3	3	4	4	2	4
2	0	0	0	4	0	0	4	4
2	4	4	4	4	4	4	4	4
2	3	3	2	1	1	2	2	2
2	1	1	1	0	0	1	0	4

2	1	1	0	0	1	1	1	2
2	1	1	0	0	0	0	1	1
2	4	3	2	3	3	3	3	4
2	1	1	1	0	1	0	1	1
2	1	0	0	0	1	1	0	1
2	3	2	3	2	2	4	3	4
2	2	1	2	3	2	3	3	3
2	3	3	3	2	3	3	2	3
2	1	1	0	0	0	0	1	1
2	3	4	3	2	3	3	2	4
2	1	0	0	0	0	1	0	0
2	2	1	0	0	0	1	1	0
2	4	3	2	1	2	2	1	2
1	4	4	4	4	4	4	4	4
2	4	3	4	3	4	4	3	4
2	1	2	1	1	2	1	1	3
2	1	1	1	2	2	3	0	3
2	3	2	1	1	3	2	1	3
2	2	1	0	0	0	1	1	1
2	1	0	0	0	1	2	1	1
2	1	1	0	0	0	1	0	1
2	3	4	2	2	4	4	3	4
1	4	4	4	3	4	4	4	4
2	1	1	0	0	1	1	0	0
2	4	4	3	3	4	3	3	4
2	1	1	0	0	1	2	0	0
2	2	2	2	1	1	2	0	1
1	4	4	3	3	4	4	4	4
2	0	0	0	0	1	1	0	0
1	3	4	4	3	4	4	4	4
2	4	4	4	3	4	4	4	4
2	3	4	2	3	3	3	3	3
2	1	1	0	0	1	1	0	1
2	3	1	0	0	1	1	0	1
2	1	1	0	0	0	1	0	1
2	1	1	0	0	0	1	0	0
2	2	2	1	1	2	2	1	2
2	2	2	1	1	1	2	1	2
2	1	1	0	0	0	0	0	1
1	2	0	0	0	1	2	0	1

2	3	4	2	2	4	4	4	4
2	2	1	1	0	1	0	2	0
1	3	3	2	2	3	3	3	4
2	2	2	1	1	2	1	1	2
1	2	1	0	0	0	1	0	1
2	1	1	0	0	0	1	0	1
2	3	2	1	1	1	2	1	2
2	1	0	0	0	1	1	0	1
1	1	0	0	0	1	1	0	1
2	1	1	0	0	1	1	0	2
2	2	2	0	0	0	1	3	0
1	4	3	3	2	3	4	4	4
1	1	1	0	0	1	0	0	1
1	1	0	0	0	0	1	0	0
1	2	0	0	0	0	1	0	1
1	3	2	1	1	1	2	1	3
1	4	2	2	2	4	4	4	4
1	3	1	2	4	4	4	4	4
1	4	3	2	3	4	4	4	4
1	4	3	3	3	4	4	3	4
2	1	1	2	1	2	1	1	1
2	1	0	0	0	1	1	0	0
2	4	4	3	3	4	4	3	4
2	4	3	3	4	4	4	4	4
2	1	0	0	0	1	1	0	1
2	4	4	3	3	4	4	4	4
2	0	0	0	0	0	0	0	2
1	4	3	2	2	4	3	3	4
1	2	2	1	0	1	2	0	2
2	1	1	0	0	0	1	0	1
2	1	0	0	0	0	1	0	1
1	2	1	0	0	0	1	0	1
1	4	3	3	3	4	4	4	4
2	1	0	0	0	0	1	1	0
1	4	3	2	2	3	4	3	4
1	4	3	3	2	3	4	3	4
1	4	3	2	3	4	4	3	4
1	4	4	3	4	3	4	4	4
2	0	0	0	0	0	0	0	4
1	2	1	2	3	2	3	1	3

1	4	3	3	2	3	2	1	2
2	3	2	1	0	1	2	1	2
2	2	0	1	0	1	2	1	2
2	1	0	0	0	1	1	0	0
2	1	1	0	0	0	1	0	1
1	1	1	0	0	0	0	1	0
1	4	3	3	3	4	4	2	4
1	4	3	4	2	3	4	3	4
1	2	1	1	0	0	1	0	2
1	2	1	1	1	1	3	3	1
1	4	3	4	3	4	4	4	4
1	2	1	0	0	1	2	1	1
2	1	0	0	0	0	1	0	0
2	2	1	0	0	0	0	0	0
2	1	0	0	0	0	0	0	0
1	1	0	0	0	0	0	0	0
1	4	4	4	3	4	4	4	4
1	0	1	0	1	0	0	1	1
1	1	0	0	0	1	1	0	0
2	1	0	0	0	0	2	0	1
1	1	0	0	0	0	1	0	1
1	1	0	0	0	0	1	0	1
1	1	0	0	0	0	2	0	1
1	4	4	3	2	4	4	4	4
1	4	4	3	3	4	4	4	4
2	4	4	4	3	3	4	2	4
2	1	0	0	0	1	1	0	2
2	4	4	3	3	4	4	4	4
2	4	4	2	3	4	4	3	4
2	4	3	4	3	4	4	4	4
2	4	2	3	2	4	4	4	4
2	1	1	0	0	1	2	0	1
1	4	3	2	3	4	4	2	4
2	1	0	0	1	1	1	0	1
2	4	4	3	2	3	4	3	4
2	3	4	3	3	4	3	3	4
2	2	1	0	0	1	2	0	1
2	4	4	3	3	3	4	4	4
2	1	0	0	0	1	1	0	1
2	0	0	0	0	0	0	0	2

2	1	0	0	0	1	2	1	2
2	4	3	3	3	2	4	4	4
2	0	0	0	0	0	0	0	2
2	0	0	0	0	0	2	0	1
2	4	4	4	3	3	4	4	4
2	0	0	0	0	0	1	0	2
2	1	1	0	0	0	1	0	1
2	1	0	0	0	0	1	0	1
1	4	3	4	4	4	4	2	3
2	1	0	0	0	0	0	1	1
2	2	2	1	1	1	2	1	1
1	4	3	3	4	3	4	3	4
1	1	2	0	1	1	2	1	1
2	0	0	0	0	0	1	0	1
1	3	4	3	3	3	4	4	4
2	1	0	0	0	1	1	0	1
1	4	3	4	4	3	4	3	4

PCS-9	PCS-10	PCS-11	PCS-12	PCS-13
3	1	3	1	2
0	0	2	0	0
3	4	3	4	3
1	1	2	1	2
3	3	4	4	4
1	0	0	1	1
0	1	2	1	0
0	0	1	2	3
2	1	2	1	2
3	3	1	1	1
3	2	3	2	2
0	0	1	1	1
3	3	3	2	2
1	2	1	2	1
0	0	0	0	0
1	1	2	0	0
3	2	2	2	1
0	1	0	0	2
3	2	3	1	3
1	4	1	2	2
0	1	1	1	1
1	1	3	1	0
1	0	2	1	1
3	2	3	3	1
1	2	3	1	1
1	3	3	1	1
1	2	3	2	1
1	2	4	3	2
4	3	4	4	2
3	0	4	0	0
2	3	4	3	2
2	4	4	0	0
1	2	2	1	1
2	2	4	3	2
2	1	4	2	2
1	2	1	1	0

2	1	3	0	1
3	2	4	2	3
2	2	4	3	2
2	2	4	2	2
2	4	4	2	2
4	1	2	3	2
1	1	0	1	1
4	2	4	3	2
3	2	4	3	1
2	2	2	1	2
3	2	4	2	3
3	2	4	3	1
2	3	3	2	1
2	2	3	2	2
0	1	2	3	0
1	2	1	1	1
0	2	3	0	0
4	4	4	4	4
2	2	4	3	2
0	0	0	0	0
1	0	2	1	1
1	2	4	2	2
1	1	1	1	1
2	2	4	3	1
3	4	3	3	3
2	3	4	4	2
2	3	4	4	2
3	4	4	3	2
3	3	3	3	3
3	4	3	3	2
2	3	4	3	2
2	3	4	2	3
2	2	4	3	2
2	2	2	2	2
3	3	3	4	2
2	2	2	2	2
1	1	3	2	1
1	1	2	1	1
0	0	0	0	0
0	1	1	1	1

2	2	4	3	4
3	4	4	3	2
2	1	3	3	1
2	3	4	3	2
3	1	4	3	2
1	1	2	1	1
3	3	4	3	2
1	1	1	0	0
3	3	3	2	2
3	3	3	3	2
3	2	4	3	2
3	3	4	2	1
2	3	4	3	2
3	3	2	4	3
2	2	3	4	2
2	3	3	3	2
3	3	4	3	2
1	2	2	2	1
0	0	2	2	1
3	3	4	3	2
1	1	2	1	1
2	2	3	2	1
2	2	4	4	3
3	2	3	2	3
3	4	4	3	4
0	1	1	1	0
2	2	4	4	2
2	3	4	3	2
2	1	0	0	1
2	2	3	2	3
2	2	3	3	2
2	2	3	2	3
2	3	4	3	1
1	1	2	3	1
4	4	2	3	4
3	4	4	3	1
0	1	1	2	1
2	1	3	3	0
3	3	4	3	2
3	3	3	3	2

1	0	2	2	0
3	3	3	3	2
2	3	4	3	2
1	0	3	2	0
1	0	1	1	0
2	2	2	3	3
1	1	4	0	1
4	4	4	3	2
0	0	0	0	0
3	3	3	2	3
1	2	1	1	0
1	4	1	2	1
0	2	0	2	0
0	1	2	0	0
3	4	4	3	2
0	0	0	0	0
2	2	2	2	2
1	2	1	2	3
1	3	4	4	2
0	1	3	1	1
3	3	3	3	3
3	3	3	3	2
0	1	0	0	0
1	1	2	1	1
3	3	3	3	3
2	2	4	3	3
3	3	3	1	1
4	4	4	3	3
0	0	0	0	0
0	0	2	1	1
3	3	2	3	3
2	4	4	3	2
3	4	3	3	2
2	2	3	3	2
2	3	4	3	2
2	3	4	4	2
0	1	4	1	1
3	4	4	4	3
1	2	2	3	1
4	1	4	0	1

1	2	2	2	1
0	0	1	0	0
3	3	4	3	2
0	0	1	0	0
0	0	1	0	0
3	4	3	3	2
3	3	3	2	3
2	3	4	3	2
0	0	1	1	0
3	3	3	2	2
0	0	0	0	0
0	1	0	0	0
1	1	2	2	1
4	4	3	3	2
2	4	4	4	2
0	1	1	2	2
0	1	1	1	1
3	2	2	1	1
0	1	1	1	0
1	1	2	2	1
1	0	1	1	0
2	3	3	4	2
3	4	4	4	1
0	0	1	0	0
2	3	3	3	3
0	0	0	1	0
0	0	2	0	0
3	3	4	3	3
0	0	1	0	0
3	4	4	4	3
3	4	4	4	2
3	3	4	3	2
0	0	0	0	0
0	0	0	1	0
0	0	0	0	0
0	0	1	0	0
0	1	1	1	0
1	1	2	2	1
0	1	0	0	0
0	0	1	0	0

3	4	4	4	3
0	1	1	0	0
2	3	4	3	2
0	1	2	1	0
0	0	1	0	0
0	0	0	1	0
0	1	2	1	0
0	1	1	1	0
0	1	2	2	0
0	1	2	1	0
1	2	2	1	0
2	3	3	3	2
0	0	0	1	0
0	0	1	0	0
0	0	2	1	0
3	3	3	3	3
3	4	4	3	3
3	4	4	4	3
3	4	4	3	2
3	4	4	4	3
0	0	1	1	0
0	0	1	0	0
4	3	4	4	3
3	4	4	3	3
0	0	1	1	0
3	4	4	3	3
0	0	0	0	0
3	4	4	3	2
0	1	1	1	0
0	0	1	0	0
0	0	1	0	0
0	0	1	1	0
2	3	4	4	2
0	0	0	0	0
3	2	4	3	1
3	3	4	3	2
3	3	4	4	2
3	4	3	3	2
1	1	3	1	0
2	2	3	3	3

2	2	3	2	1
0	0	1	1	1
1	1	3	1	1
1	0	1	1	0
0	0	1	1	0
1	0	1	1	1
3	3	4	3	2
3	3	4	3	2
0	1	2	0	0
2	1	2	1	0
3	2	4	4	2
1	1	2	2	1
0	0	1	0	0
0	0	1	0	0
0	0	0	1	0
0	0	0	0	0
3	4	4	3	2
1	2	3	1	1
0	0	0	1	0
0	0	2	1	0
0	0	1	0	0
0	0	1	0	0
0	0	1	0	0
3	4	3	4	2
4	3	4	4	2
3	4	4	3	2
1	2	3	2	0
3	4	4	4	2
3	4	4	3	2
3	3	4	3	2
3	4	4	3	2
0	0	1	1	0
3	3	4	3	2
0	0	1	0	0
2	4	4	3	2
2	2	4	3	2
0	1	1	1	0
3	4	4	3	2
0	0	1	1	0
0	0	1	1	0

0	0	1	1	0
2	3	3	3	2
0	0	1	0	0
0	0	1	1	0
3	3	4	3	2
0	0	1	0	0
0	0	1	1	0
0	0	1	1	0
3	4	4	3	2
0	0	0	0	0
0	0	1	1	0
2	3	4	2	2
1	1	2	1	1
0	0	1	0	0
3	2	3	3	2
0	0	1	0	0
3	4	4	3	2
